# New insights into the role of endosomal proteins for African swine fever virus infection

**DOI:** 10.1371/journal.ppat.1009784

**Published:** 2022-01-26

**Authors:** Miguel Ángel Cuesta-Geijo, Isabel García-Dorival, Ana del Puerto, Jesús Urquiza, Inmaculada Galindo, Lucía Barrado-Gil, Fátima Lasala, Ana Cayuela, Carlos Oscar S. Sorzano, Carmen Gil, Rafael Delgado, Covadonga Alonso

**Affiliations:** 1 Departmento de Biotecnología, INIA-CSIC, Centro Nacional Instituto Nacional de Investigación y Tecnología Agraria y Alimentaria, Madrid, Spain; 2 Instituto de Investigación Hospital 12 de Octubre Imas12, Madrid, Spain; 3 Centro Nacional de Biotecnología CSIC, Madrid, Spain; 4 Centro de Investigaciones Biológicas Margarita Salas CSIC, Madrid, Spain; 5 Facultad de Medicina, Universidad Complutense de Madrid, Madrid, Spain; Institute of Molecular Biology, Academia Sinica, TAIWAN

## Abstract

African swine fever virus (ASFV) infectious cycle starts with the viral adsorption and entry into the host cell. Then, the virus is internalized via clathrin/dynamin mediated endocytosis and macropinocytosis. Similar to other viruses, ASF virion is then internalized and incorporated into the endocytic pathway. While the endosomal maturation entails luminal acidification, the decrease in pH acts on the multilayer structure of the virion dissolving the outer capsid. Upon decapsidation, the inner viral membrane is exposed to interact with the limiting membrane of the late endosome for fusion. Viral fusion is then necessary for the egress of incoming virions from endosomes into the cytoplasm, however this remains an intriguing and yet essential process for infection, specifically for the egress of viral nucleic acid into the cytoplasm for replication. ASFV proteins E248R and E199L, located at the exposed inner viral membrane, might be implicated in the fusion step. An interaction between these viral proteins and cellular endosomal proteins such as the Niemann-Pick C type 1 (NPC1) and lysosomal membrane proteins (Lamp-1 and -2) was shown. Furthermore, the silencing of these proteins impaired ASFV infection. It was also observed that NPC1 knock-out cells using CRISPR jeopardized ASFV infection and that the progression and endosomal exit of viral cores was arrested within endosomes at viral entry. These results suggest that the interactions of ASFV proteins with some endosomal proteins might be important for the membrane fusion step. In addition to this, reductions on ASFV infectivity and replication in NPC1 KO cells were accompanied by fewer and smaller viral factories. Our findings pave the way to understanding the role of proteins of the endosomal membrane in ASFV infection.

## Author summary

African swine fever virus (ASFV) causes a deadly disease of pigs and wild boars that was endemic in Africa but has spread in recent years to Europe, Asia and Oceania with a high socioeconomic impact. ASFV enters the cell by endocytosis and has adapted to the endosomal conditions to acquire infectivity. Fusion of the internal viral membrane with the endosomal membrane is required for the exit of viral DNA into the cytoplasm to start replication. We have found that ASF virion internal membrane proteins E248R and E199L interact with the endosomal proteins Niemann Pick C1 (NPC1) and lysosomal membrane proteins (Lamp)-1 and -2. And, appear to be required for endosomal trafficking of ASF virions endosomal traffic and exit to the cytoplasm in the cell entry process. These molecules act regulating cholesterol flux from the endosome to the endoplasmic reticulum, and appear to be important for the viral infection cycle. In silenced and knockout cells, ASFV infection was affected at early and later stages. In null cells, virion entry and progression through the endosomal pathway at entry was arrested and several viral cores were retained at late endosomes without entering the fusion phase for cytoplasmic exit. These results provide new insights into the role of endosomal proteins for ASFV infection.

## Introduction

African Swine Fever Virus (ASFV) is the only known member of the *Asfarviridae* family, and the only known DNA arbovirus. It is a large, enveloped virus with an average diameter of 200 nm and a multilayered structure and icosahedral morphology that has been recently unveiled in detail [1–3].

ASFV is the causative agent of the high mortality haemorrhagic disease affecting swine that is endemic in sub-Saharan Africa. However, ASF epidemics that started in the Caucasus and Russian Federation in 2007 [4], have rapidly spread to different countries in Europe, Asia and Oceania; causing a devastating burden on the global pig industry [5]. Currently, ASF cases have been reported in Germany since September 2020 [6]; in addition to this, the Dominican Republic has also recently declared an outbreak of ASF [7–9].

ASFV infectious cycle starts with the viral adsorption and entry into the host cell. After attachment to an unknown receptor, the virus is mainly internalized via clathrin/dynamin mediated endocytosis and macropinocytosis [10,11]. Then, the virion is internalized and incorporated into the endocytic pathway.

Under the molecular cues of endosomes, the multi-layered ASF virion undergoes uncoating starting from decapsidation [12]. This step will be followed by a poorly characterized fusion process at late endosomes (LE), that conveys the delivery of the naked viral core into to the cytoplasm [13].

Previous studies have demonstrated that the endosomal maturation entails dropping of luminal pH and this acidic environment disrupts the structure of the ASF virion producing decapsidation at 30–45 minutes post infection (mpi) [12]. As a consequence, the exposure of the following layer, the inner viral membrane would allow its fusion with the limiting membrane of the LE [13]. Cholesterol efflux at the LE is relevant at this stage and, its pharmacological blockade at this level can cause retention of virions inside endosomes, and inhibits infection progression [14]. Endosomal cholesterol efflux is mainly regulated by the coordinated action of the NPC1, an endolysosomal membrane protein; and NPC2, a single domain protein located in the lumen of the vesicle [15–17]. Also, other endolysosomal molecules such as Lamp1 and Lamp2, cooperate with cholesterol flux at the endosomal membrane [18–20].

It has been demonstrated the importance of both NPC1 and cholesterol transport for a successful infection of a variety of viruses [21–32]. An example is Ebola virus (EBOV), in which NPC1 has a pivotal role at infection, given that the EBOV Glycoprotein (GP) interacts with NPC1 [33] to achieve endosomal fusion and cytoplasmic penetration. The disruption of this interaction by drugs that are known to interact with NPC1, causes a reduction in the viral replication, and it is currently accepted that NPC1 is pivotal for a successful infection in several virus models [34,35]. Another example is Lassa virus, an Arenavirus, which interacts with another endolysosomal protein, Lamp1 [36]. In addition to this, NPC1 has been recently shown to be important for SARS-CoV-2 [32,37]. All these studies suggest the importance of the NPC1 protein for several distant viruses.

Since the underlying mechanisms of ASFV viral fusion and egress to the cytoplasm remain still obscure, we hypothesized that this interaction could be relevant for this virus in order to penetrate to the cytoplasm from LE in a NPC1-dependent manner via proteins belonging to its fusion machinery.

The ASFV proteins potentially involved in fusion step, would be viral proteins E248R and E199L, which are structural proteins located at the virion internal membrane. These proteins have been postulated to be implicated in the ASFV fusion step in order to release the naked viral core from the LE to the cytoplasm [13,38,39]. Therefore, these viral fusion proteins could potentially interact with endosomal membrane proteins for fusion.

Furthermore, E248R and E199L weakly resemble to different subunits of the fusion complex of poxvirus, making them suitable candidates to exert their function at the endosomal exit and cytoplasmic penetration of viral cores. ASFV E248R is a type II late structural protein belonging to a class of myristoylated proteins related to the vaccinia virus (VACV) L1R protein, which is part of the VACV fusion complex. ASFV E199L is a transmembrane protein type I, which resembles three subunits of the poxviral fusion machinery, A16, G9 and A26 [40,41].

In this study, we have reported ASFV E248R and E199L proteins as a potential interactors of the endosomal proteins NPC1, Lamp1 and Lamp2. This study brings to the forefront the endosomal membrane proteins as relevant targets to counteract the early steps of the ASFV infection.

## Results

### Cholesterol accumulation is critical for ASFV infection

**Fig 1:**
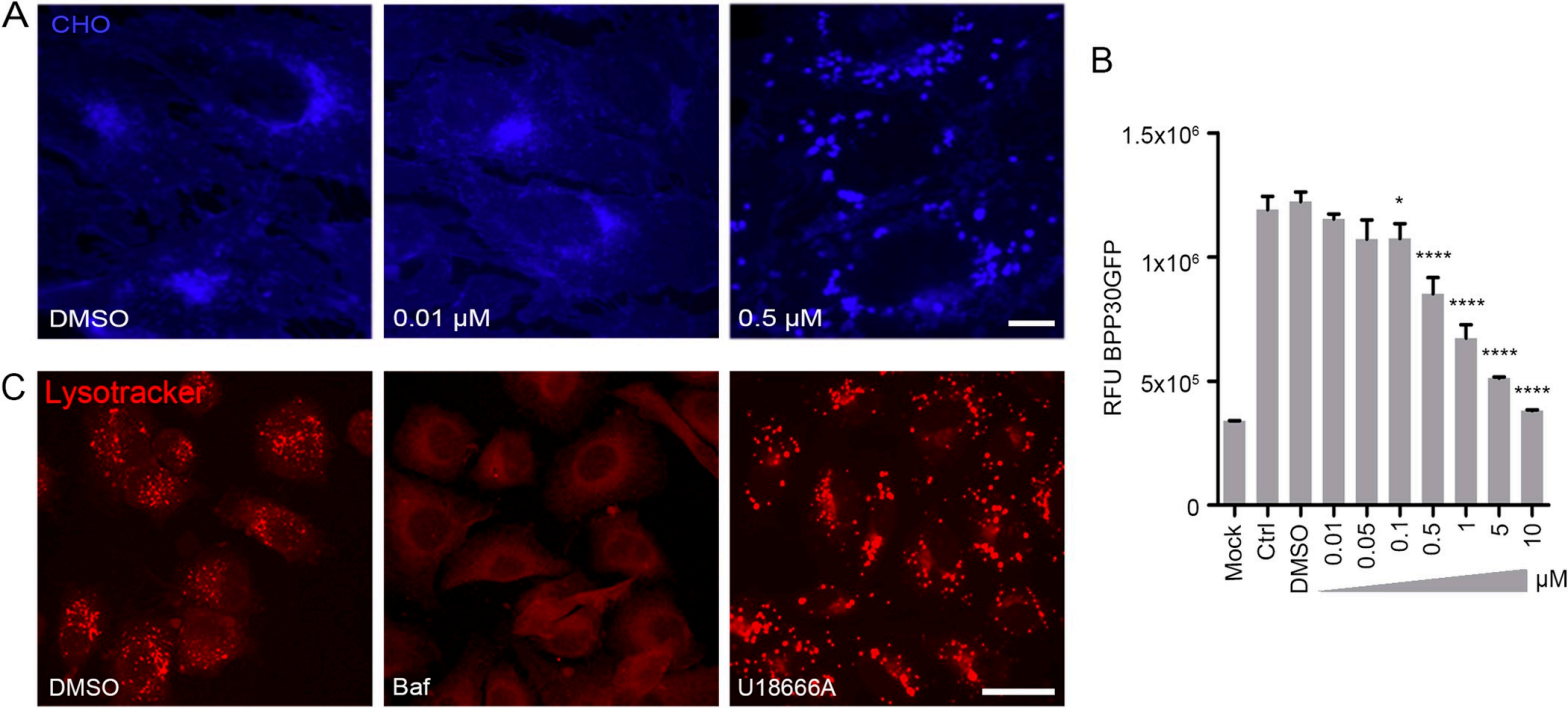
Cholesterol efflux arrest and inhibition of ASFV infectivity. (A) Treatment with increasing concentrations of U18666A chemical compound altered the distribution of unesterified cholesterol (CHO) in Vero cells (filipin; blue). Scale bar: 10 μM. (B) Cells pre-treated with increasing concentrations of U18666A compound and infected with recombinant ASFV BPP30GFP for 16 hpi. GFP fluorescence intensity was measured using a plate reader. Graph represents mean±sem of 3 independent experiments. Statistically significant differences are indicated by asterisks (****p<0.0001, *p<0.05). (C) Visualization of acidic vesicles with lysotracker (red) in Vero cells treated with U18666A drug or controls. These acidic vesicles were absent after treatment with lysosomotropic drugs (Scale bar: 25 μM).

To determine the influence of cholesterol flux on ASFV infection, the chemical U18666A, a compound that blocks the cholesterol transporter NPC1 [14] was used. This compound reduces cholesterol efflux from the endosome; thus, cholesterol is retained within these vesicles and its accumulation resulted in the formation of large, dilated endosomes. To further study the effect of this on ASFV infection, we explored whether there was a correlation between cholesterol retention and reduced ASFV infectivity at the same drug concentrations. For this purpose, Vero cells were pre-treated with increasing concentrations of the drug U18666A and cholesterol was labelled with Filipin, a compound that binds to cholesterol. As can be seen in Fig 1A, a characteristic accumulation of cholesterol in large vesicle-like clusters around the perinuclear zone was observed from a drug concentration of 0.5 μM. Similar results were observed when Vero cells were pre-treated with similar concentrations of drug U18666A and infected with ASFV BPP30 GFP at a multiplicity of infection (MOI) of 1 pfu/ml for 16 hpi. After this time, GFP expression was measured using a plate reader. It was observed that infection started to be impaired at the same drug concentrations that cholesterol accumulation started, suggesting that there might be a correlation between the two events (Fig 1B). To further confirm that this was not a side effect of the drug U18666A on acidification; the effect of acidification under various conditions, including the cholesterol efflux blocking drug U18666A, was analysed using the Lysotracker acid pH probe [12]. As controls, Vero cells were pre-treated with the lysosomotropic drugs Bafilomycin-A1 (Fig 1C) and NH_4_Cl (not shown). Acidic vesicles stained clearly in controls and in cells treated with drug U18666A, whereas no signal from acidic vesicles was detected with the lysosomotropic drugs. This confirmed that compound U18666A acted specifically as an inhibitor of cholesterol export in this context (Fig 1C).

Therefore, we set out to investigate the potential interaction of the E248R and E199L proteins of ASFV with one of the endosomal protein responsible for cholesterol trafficking, NPC1, and also its possible role in ASFV infection.

### Interaction of ASFV E248R and E199L proteins with NPC1

Some studies suggest a possible role of ASFV E248R and E199L proteins in membrane fusion [13]. These proteins are highly conserved among ASFV isolates (S1A and S1B Fig). In addition, both proteins resemble in their structure several subunits of the poxvirus multiprotein entry/fusion complex (S1C and S1D Fig).

To elucidate the potential role of ASFV E248R and E199L proteins in viral fusion in the endosome, we investigated whether these viral proteins could interact with endosomal proteins such as NPC1, NPC2, Lamp1 and 2.

For this purpose, constructs were designed for the expression of E248R and/or E199L proteins tagged to a reporter protein (EGFP). These proteins were codon optimized and tagged to EGFP at the N-terminus and expressed as EGFP-fusion proteins in HEK 293T cells. An empty vector expressing EGFP was used as a control.

For this study, HEK 293T cells were selected because of their high transfection efficiency as well as being the cell line of choice for protein-protein interaction studies of various viruses [42].

**Fig 2:**
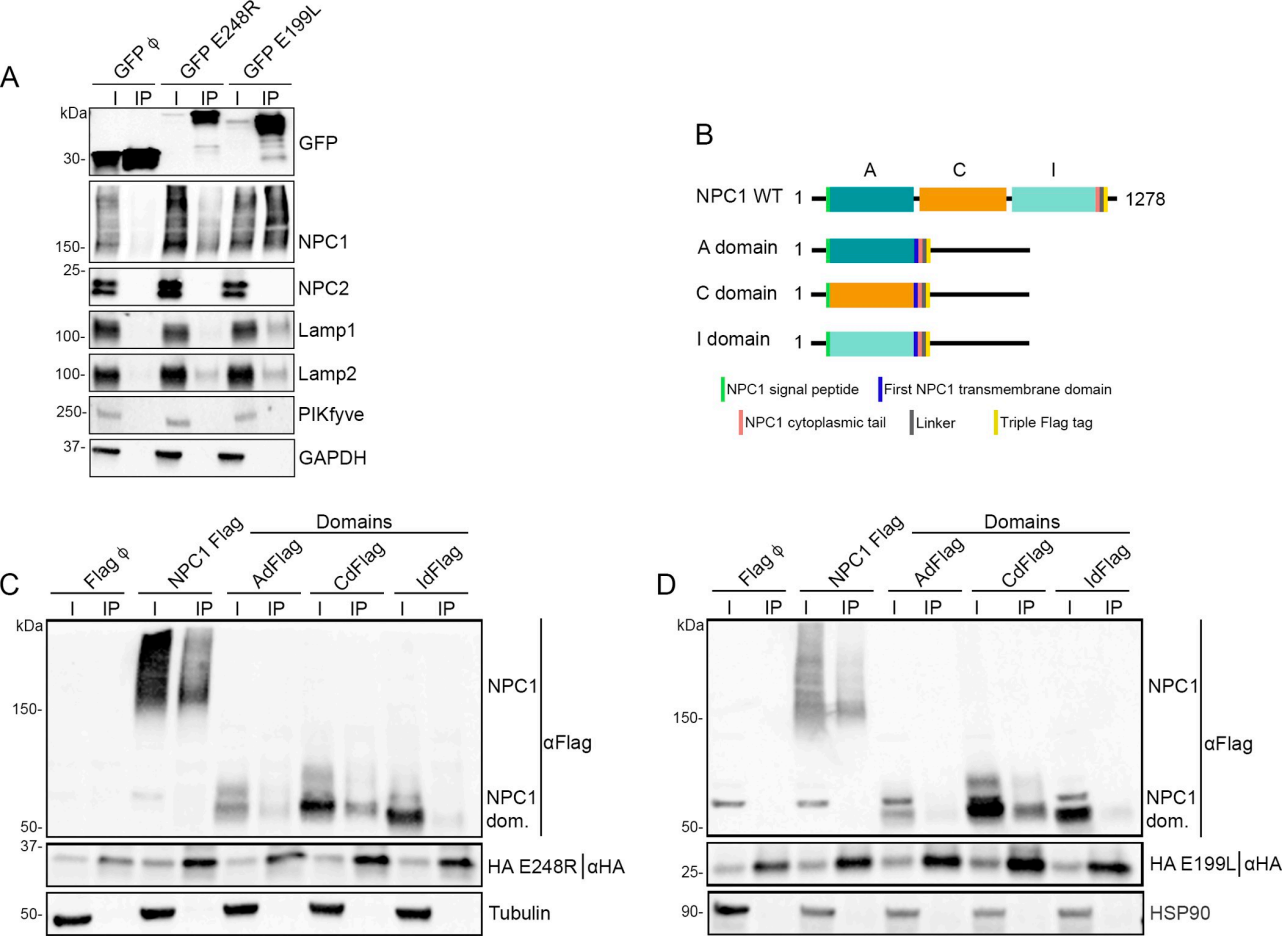
E248R and E199L interactions with endosomal proteins NPC1, Lamp 1 and Lamp2. (A) GFP was immunoprecipitated in lysates from HEK293T cells transfected with EGFP, EGFP E248R or EGFP E199L. Representative immunoblot analysis of cell lysates (I) and GFP-immunoprecipitates (IP) using GFP, NPC1, NPC2, Lamp1, Lamp2 and PIKfyve antibodies. GAPDH was used as control. (n  =  4 independent experiments, S2 and S3 Figs). (B) Schematic representation of NPC1 WT and specific recombinant constructions of individual domains shown in C and D. (C, D) HA was immunoprecipitated in lysates from HEK293T cells co-transfected with HA E248R (C) or HA E199L (D) with NPC1 Flag and FLAG NPC1 domains. Representative immunoblot analysis of total lysates (I) and HA-immunoprecipitates (IP) using Flag and HA antibody. Alpha-tubulin (C) or HSP90 (D) were used as control. “NPC1 dom.” refers to individual domains of NPC1 (n  =  3 independent experiments, S5 and S6 Figs).

Next, EGFP-tagged viral proteins, E248R and EGFP E199L were immunoprecipitated using EGFP-Trap system beads (Chromotek). After immunoprecipitation, both input samples (cell lysate) and bound samples (elution sample) were analysed by western blot (WB) (Fig 2A). As it is shown in Fig 2A, both viral proteins (E248R and E199L) were found to interact with NPC1 and Lamp2. However, Lamp1 was only co-precipitated with E199L and endosomal proteins PIKfyve and NPC2 were not co-precipitated with either of the ASFV proteins. Cellular protein GAPDH was used as a load and negative control.

### E248R and E199L interaction with NPC1 domains

To identify the specific NPC1 domain involved in the interaction with E248R and E199L, three constructs expressing individual NPC1 domains (A, C and I) fused to Flag were designed. These NPC1 domains were the NPC1 domains defined by Miller et al., 2012 (Fig 2B) [43]. Each NPC1 domain was co-expressed together with HA E248R for 24 hours in HEK 293T cells. After cell lysis, immunoprecipitation of the samples was performed using protein G beads and HA-specific monoclonal antibodies (Fig 2C). Expression of HA E248R and individual domains was then confirmed using anti-HA and anti-Flag antibodies.

Both the NPC1 WT and C domain were retrieved in the bound samples obtained from Co-IP and analysed by WB using an anti-Flag antibody. These results showed that the C domain of NPC1 is required for the interaction (Fig 2D).

Simultaneously, we were also able to demonstrate an interaction between VACV L1R and NPC1-Flag (S1E Fig). In contrast, another independent HA-fused cellular protein HA-eIF4E (S4B Fig), used as a negative control, was not immunoprecipitated, corroborating the specificity of interaction between HA E248R and HA E199L with full length NPC1 and the C-domain of NPC1.

### Silencing of NPC1 and Lamp2 protein expression impaired ASFV infection

**Fig 3:**
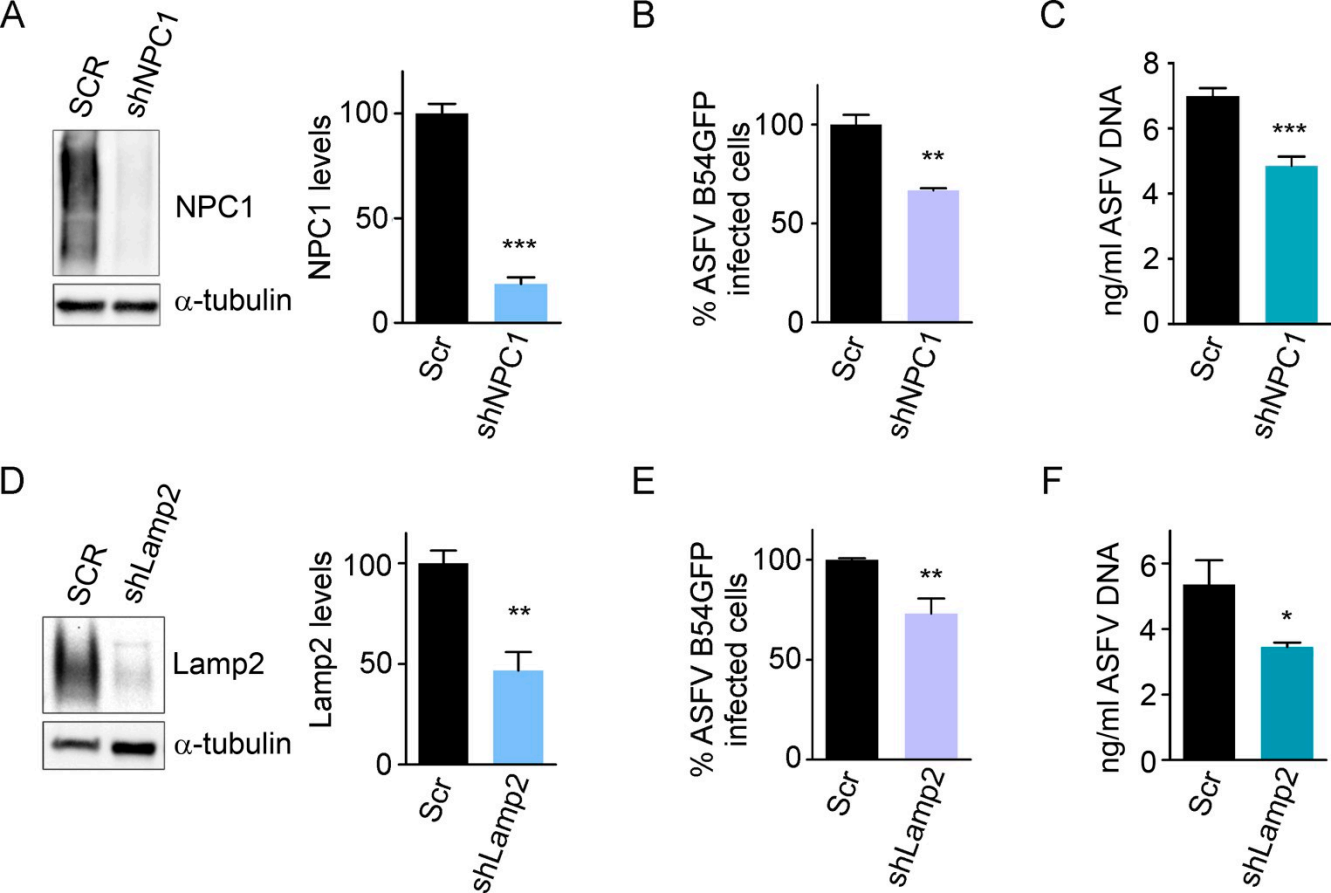
Silencing of NPC1 and Lamp2 proteins affected ASFV infection. (A, D) Representative immunoblots and densitometric analysis of NPC1 (A) and Lamp2 (D) in Vero cells transduced with lentiviral particles encoding NPC1 and Lamp2 shRNAs or Scrambled shRNA (SCR) as controls. Αlpha-tubulin was used as loading control. (B, E) Flow cytometry of cells shown in (A, D) and infected with fluorescent recombinant virus B54GFP at a MOI of 1pfu/ml for 16 hpi. (C, F) Quantification of ASFV DNA by qPCR in cells shown in (A, D). Graphs represent mean±sem of three independent experiments. Statistically significant differences are indicated by asterisks (***p < 0.001, **p < 0.01, *p < 0.05).

Silencing the expression of these potential cellular targets for ASFV fusion proteins was achieved in search for their functionality using shRNA technology as described below. Validation of silencing was achieved by demonstrating the absence of protein expression for NPC1 or Lamp2 in WB and quantified by densitometry (Fig 3A and 3D). NPC1 shRNA, Lamp2 shRNA and scrambled (SCR) shRNA Vero cells were infected with recombinant fluorescent virus (B54GFP), which expresses GFP as a fusion of the major infection protein p54, at a MOI 1 pfu/ml to infect cells. Silencing of NPC1 and Lamp2 reduced the percentage of infected cells compared SCR cells when measured by flow cytometry at 16 hpi (Fig 3B and 3E). A reduction of ASFV replication, quantified by qPCR was also observed in these cells (Fig 3C and 3F).

The first evidence obtained for a potential role of NPC1 in ASFV infection was the inhibition observed in Vero cells treated with various inhibitor compounds targeting the interaction of EBOV GP with NPC1 [44]. This prompted us to investigate the possible interaction of NPC1 with ASFV viral proteins. Similar to this study, the inhibitory effect of the same compounds was tested in primary porcine macrophages at the following doses SC073 100 μM, SC816 5 μM and SC198 50μM (S7 Fig).

To determine the non-cytotoxic working concentrations of the different compounds and the vehicle control (organic solvent DMSO), the CellTiter 96 Non-radioactive Cell Proliferation Assay (Promega) was used (S7A Fig). Based on these results, the optimal non-toxic working concentrations for the compounds were selected for the infection assays and viral DNA replication was examined by qPCR as described below.

Primary porcine alveolar macrophage cultures were also tested and infected with BA71V ASFV at a MOI of 1 pfu/ml for 16 hpi and, it was found that compounds SC198 and SC073 had a significant inhibitory effect on ASFV replication, as shown in S7B Fig. With this experiment, we could ascertain that the same inhibitors targeting the EBOV GP/NPC1 interaction, were also actively inhibiting ASFV replication in porcine primary macrophages.

### Validation of NPC1 Knock-out cells

After confirming the interaction of viral proteins with NPC1, and to study the possible role of NPC1 in ASFV infection, we tested the impact of the absence of this cellular protein on the infection. To this end, we generated a Vero cell line knockout (KO) for NPC1 using CRISPR-Cas9 technology as described in Materials and Methods. The absence of NPC1 was confirmed in the KO cells by WB and indirect immunofluorescence (IFI) (S8A and S8B Fig).

The absence of NPC1 resulted in the accumulation of cholesterol in enlarged acidic vesicles, as it can be observed in S8C Fig. A similar cholesterol accumulation pattern was observed in cells pre-treated with compound U18666A compared to untreated (S8D Fig).

An additional experiment with EBOV infection was used to validate the NPC1 KO cells. As NPC1 acts as an intracellular receptor for EBOV, NPC1 KO cells should be resistant to infection with this virus as previously reported [33]. This was verified by infection with a vesicular stomatitis virus (VSV) pseudotyped with the glycoprotein (GP) of the Mayinga strain of EBOV, confirming the functional abrogation of NPC1 in these KO cells S8E Fig).

**Fig 4:**
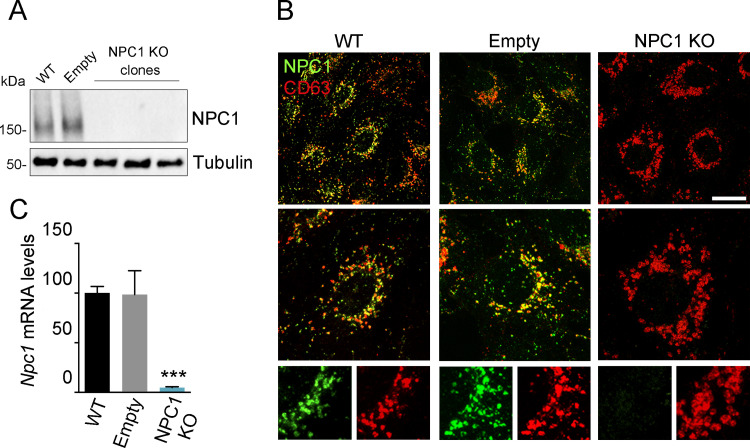
Validation of CRISPR KO NPC1 cells. (A) Immunoblot of NPC1 and tubulin (loading control) in samples from WT, Empty and clones of NPC1 KO cells. (B) Visualization NPC1 (green) in late endosomes (CD63, red) in WT, Empty and NPC1 Vero KO cells. Scale bar: 25 μm. Zoom images are also shown. (C) Npc1 mRNA levels in WT, Empty and NPC1 KO cells as detected by qPCR. Graph represents mean±sem of three independent experiments. Statistically significant differences are indicated by asterisks (***p < 0.001).

After confirming the absence of NPC1 protein expression by WB in several clones from the KO cell pool, we selected a single clone, hereafter referred to as NPC1 KO from now on (Fig 4A). In addition, we checked for NPC1 knock out in the selected clone by IFI (Fig 4B). Finally, we checked the lack of Npc1 mRNA in these NPC1 KO cells by RT-qPCR (Fig 4C). NPC1 expression remained unchanged in parental WT Vero cells and the CRISPR-selected control Vero cells (LCV_2_ 1 clone, named Empty) (Fig 4).

Once the absence of NPC1 protein in the KO cells was confirmed, their susceptibility to ASFV infection was investigated by analysing early ASFV infection, viral replication, and viral factory formation.

### NPC1 KO triggered viral cores retention inside endosomes at penetration step

**Fig 5:**
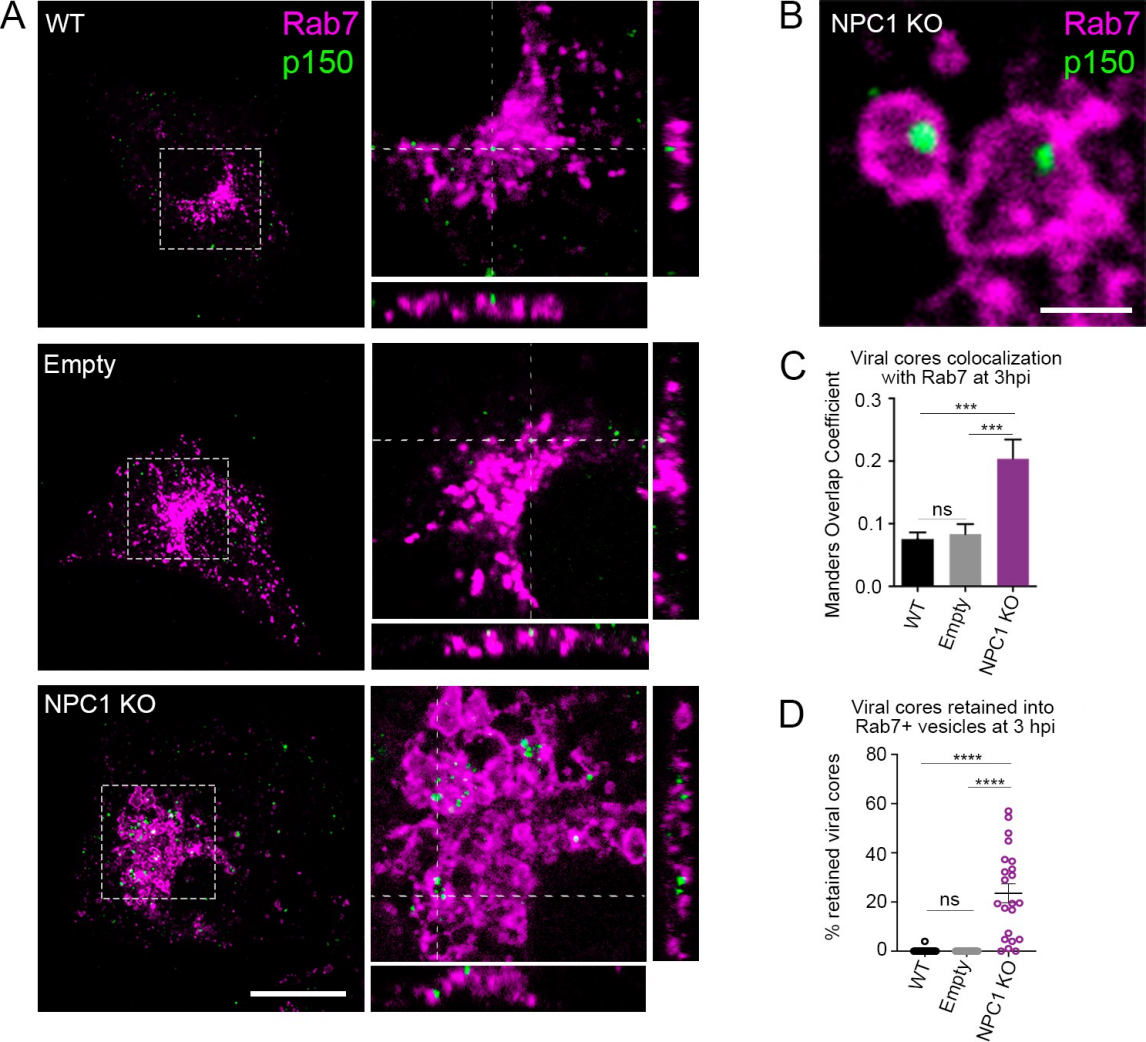
Viral cores visualized inside dilated late endosomes in ASFV infected NPC1 KO cells. (A) Representative micrographs of control cells (WT and Empty) and NPC1 KO cells infected at a MOI of 50 pfu/ml for 3hpi. Viral cores and late endosomes were detected with an antibody against p150 (green) and Rab7 (magenta), respectively. Orthogonal projections showed viral cores and Rab7 positive membranes in the Z stack. Note that numbers of retained viral cores were higher in NPC1 KO cells compared to controls. Scale bar: 10μm. (B) Insets depict endosomes containing viral cores shown in detail. Scale bar: 2 μm. (C) Overlapping Manders coefficient (mean±sem) from 2 independent experiments (n = ca. 25). (D) Percentages of viral cores inside endosomes (mean±sem) from 2 independent experiments (n = ca. 25). Statistically significant differences are indicated by asterisks (****p < 0.0001, ***p < 0.001, ns-not significative).

Previous studies have shown that in the early stages of infection ASFV traffics to the LE for decapsidation, viral fusion, and final penetration of cores into the cytoplasm [12,13]. Therefore, we wondered whether NPC1 might exert a role in the egress of the viral cores into the cytoplasm at the fusion step. To this end, we infected Vero WT, empty control and NPC1KO cells and fixed the cells after 3 hpi, as it is known that at this time point most viral cores exit LE to the cytoplasm proceeding with infection [12,13]. Next, virion trafficking through the LE was studied by immunofluorescence, in which viral cores and LE were detected using antibodies against viral protein p150 and the cellular protein Rab7 respectively. A remarkable accumulation of ASFV viral cores was observed within LE compared to controls, where, as expected for fluid endosomal traffic, only a discrete association of viral cores and LE could be observed (Fig 5A and 5B). Colocalization between viral cores and Rab7 (labelled LE) increased in NPC1 KO cells early after infection (Fig 5C). However, the numerous LE-trapped virions observed in confocal images (events) suggested that colocalization values likely underestimated the number of events (Figs 5B and S10). To test this, we created and ran a Plugin for Image J as a method to quantify viral cores retained in endosomes following the procedure described in detail in Materials and Methods (S9 Fig). A high number of viral cores retained within the LE was observed in NPC1 KO cells compared to the controls (Fig 5D). These results showed the impact of the absence of NPC1 in the process of viral fusion and egress into the cytoplasm at early stages of the infection.

### ASFV infection in NPC1 KO cells at late times

**Fig 6:**
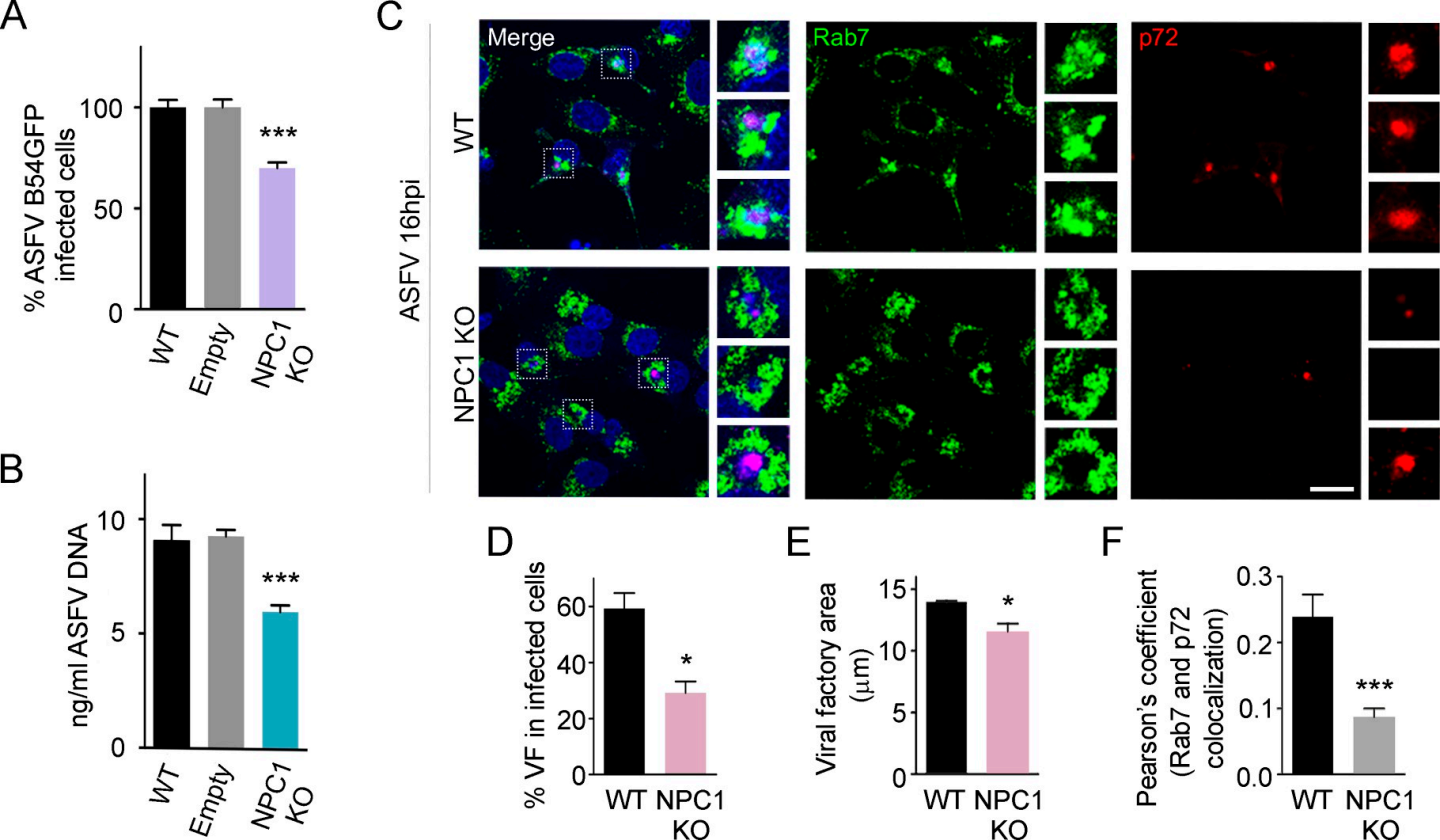
ASFV infection and replication in NPC1 KO cells. Percentage of B54GFP infected cells (1 pfu/ml) at 16hpi in WT, Empty and NPC1 KO Vero cells detected by flow cytometry. Percentages were normalized to values in WT cells. (B) ASFV replication in WT, Empty and NPC1 KO ASFV infected cells quantified by real-time PCR. (C) Representative confocal images of WT and NPC1 KO cells stained for Rab7 (green), ASFV p72 (red) and DNA (Topro3, blue). Scale bar: 20 μm. Zoom images of ASFV viral factories (boxed regions) are also shown. (D) Percentages of viral factories in WT and NPC1 KO in infected cells shown in C. (E) Quantification of the cellular area occupied by viral factories stained with ASFV p72 in WT versus NPC1 KO infected cells shown in C. (F) Quantification of Rab7 and p72 colocalization in WT and NPC1 KO ASFV infected cells using ImageJ software. Graphs represent mean±sem from three independent experiments. Statistically significant differences are indicated by asterisks (***p < 0.001, **p < 0.01, *p < 0.05).

After analysing the impact of the absence of NPC1 in ASFV entry, we also tested its impact in later stages of infection. To do this, we infected control and NPC1 KO Vero cells with ASFV B54GFP at a MOI of 1 pfu/ml for 16 hpi and quantified the number of infected cells by flow cytometry. A 30–35% reduction of ASFV infected cells was observed in NPC1 KO cells compared to controls (Fig 6A). Also, viral replication was analysed by qPCR and a significant decrease in viral replication was obtained in NPC1 KO cells compared to controls (Fig 6B). Vero WT parental cells and cells transduced with empty vector (Empty) were used as controls. Having verified that the infectivity and replication parameters were the same in both controls, we selected the WT as a control from now on in the following experiments.

In addition, we studied viral replication sites or viral factories (VF) using fluorescence microscopy with antibodies against the ASFV major protein p72 and Rab7. As expected, the major viral protein p72 accumulated in VF together with viral DNA (Fig 6C, ASFV infected panel) presenting a characteristic compact appearance as large perinuclear aggregates. However, in NPC1 KO-infected cells, fewer number of cells were harbouring VF (Fig 6D) or existing ones were altered or reduced in size (Fig 6E) compared with the control.

Furthermore, Rab7-labelled late endosomes were found in aggregates around p72-labelled VF (Fig 6F) as it has been previously described [45]. In addition to this, Rab7-positive membranes appeared to be loosely distributed in NPC1 KO cells compared to the closer contact with the VF observed in controls, as indicated by the reduced colocalization percentages (Fig 6F).

### The potential role of NPC1 and NPC2 in ASFV infection

**Fig 7:**
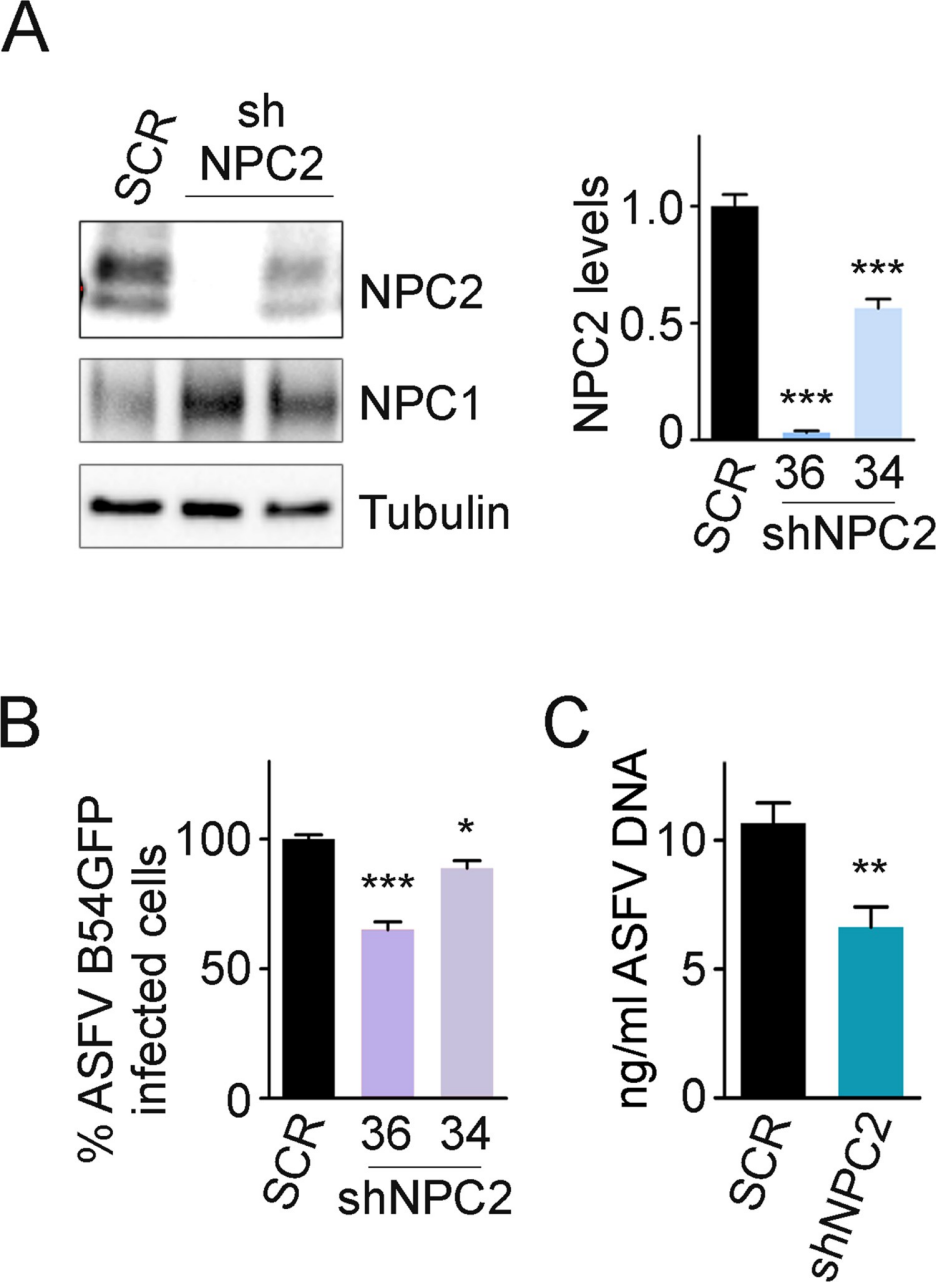
NPC2 downregulation reduced ASFV infectivity. (A) NPC1, NPC2 and tubulin (loading control) were detected by western blot in Vero cells transduced with SCR (scramble) and shNPC2 (34 and 36) lentiviral particles. Quantification of NPC1 and NPC2 bands were corrected to tubulin data and then normalized to SCR values. (B) Percentage of B54GFP ASFV infected cells (1 pfu/ml) at 16 hpi in Vero cells shown in (A). Percentages were normalized to values in SCR cells. (C) Quantification of ASFV replication in SCR and shNPC2-36 transduced Vero cells by qPCR. Graphs represent mean±sem from three independent experiments. Statistically significant differences are indicated by asterisks (***p < 0.001, **p < 0.01, *p < 0.05).

NPC2 is a single-domain luminal endosomal protein that works cooperatively with NPC1 to regulate the exit of endocytosed cholesterol from the late endosome [17,46–49]. Despite of the absence of an obvious interaction by co-immunoprecipitation, we investigated whether its cooperation with NPC1 might also have a role in ASFV infection. To this end, we used a shRNA technology to silence NPC2 expression. Lentiviral particles encoding two different shRNA sequences (denoted 34 and 36) were generated for NPC2 silencing in Vero cells; a scrambled (SCR) shRNA sequence was used as a control. The absence of NPC2 expression was confirmed by WB (Fig 7A). The percentages of infected cells (B54GFP 1 pfu/ml, 16 hpi) assessed by flow cytometry were lower in silenced NPC2 cells compared to the control (Fig 7B). Similar results were found when ASFV replication was quantified by qPCR (Fig 7C).

Taken together, these results support that the cooperative action of cellular proteins NPC1 and NPC2 could be important for ASFV infection focusing the importance of late endosomal proteins in ASFV replication cycle.

## Discussion

African swine fever (ASF) is an economically important disease of domestic swine and wild boar for which no vaccines or treatment is available. Infectious entry of ASFV relies on the trafficking of virions through the endocytic pathway in an orchestrated multi step process that depends on several molecular cues, characteristic of endosomes. However, information is lacking on the exact mechanisms, including information of the virus and host proteins involved in this process. Viral uncoating begins with an acidification-dependent step in the LE, a crucial compartment for infection [12]. The acidic environment of the late endosome is capable of dissolving the viral capsid composed of major capsid protein, p72. Decapsidation allows subsequent exposure of the inner layers, necessary for further infection events. Once the inner viral lipid membrane under the capsid is exposed, it would eventually fuse with the LE limiting membrane [50].

ASFV proteins potentially involved in fusion are localized to the inner viral membrane, such as the E248R and E199L fusion proteins. These proteins are highly conserved among ASFV isolates and share certain degree of homology and conserved structural motifs with VACV fusion complex proteins, E248R with the L1R, whereas ASFV E199L resembles VACV A16, A26 and G9, respectively [40]. This comparison is important given that VACV is a virus closely related to ASFV.

E248R is a type II myristoylated transmembrane protein. It is composed of an N-terminal portion of 199 amino acids, which contains four cytoplasm-facing cysteine residues, a hydrophobic helical transmembrane domain of 21 amino acids, and an extracellular region of 28 amino acids. E248R could contain intramolecular disulphide bonds [51]. In fact, the distribution of cysteine residues in E248R (S1C Fig), as compared to the related VACV L1R protein would suggest that the two disulphide bonds of L1R between amino acids 28–62 and 122–156 might be also present in E248R [51].

E199L is a type I transmembrane protein, with a large N-terminal cysteine enriched portion oriented internally to the viral particle, and small transmembrane and external (C-terminal) domains. E199L shares some degree of homology to three cysteine enriched proteins belonging to the fusion machinery of VACV named A16, A26 and G9, which also form disulphide bonds [52] as shown in S1D Fig.

Moreover, E248R and E199L belong to an ortholog cluster of genes of members of the family of nucleocytoplasmic large DNA viruses (NCLDV), sharing these structural motifs conserved in several viruses of this broad family, which could indicate a high functional importance of these proteins.

The entry/fusion process of ASFV occurs in the endosomes of the LE compartment and viral fusion would result in the egress of the naked cores into the cytoplasm [12,38]. Therefore, certain endosomal proteins could be involved in this fusion step, as occurs with other viruses such as EBOV [33] and Lassa virus [36].

It was previously shown that disruption of cholesterol efflux results in secondary cholesterol accumulation inside endosomes, which can partially inhibit ASFV infection [14]. Complementing our previous studies, here we found that both events were affected at similar concentrations of the compound U18666A, which inhibits the activity of the NPC1 transporter, suggesting that both events are closely linked (Fig 1).

In this context, we searched for proteins that would interact with candidate ASFV fusion proteins, namely E248R and E199L. These two highly conserved ASFV proteins, located in the internal membrane of the virion, would be exposed after decapsidation in the LE. Indeed, E248R has been suggested to be involved in viral membrane fusion and core delivery but not in viral disassembly within endosomes. Similarly, E199L was required for viral core penetration and was reported to be unrelated to downstream processes [13,39,53]. This suggests that their functions might be complementary but not identical. Likewise, proteins belonging to the VACV fusion complex are crucial for DNA replication but not for morphogenesis [41,54].

The ASFV E199L protein was characterized as a virion protein that is expressed late after infection and localizes to the virus assembly sites [52]. E199L has been reported to be a positive regulator of the NLRP3- (NLR Family Pyrin Domain Containing 3) and AIM2- (Absent in melanoma 2) inflammasome mediated inflammatory response [55]. It has also been reported to be an important antigenic protein in immunoassays [56]. In addition to these functions, in the absence of E199L, ASFV infection drastically decreased and viral particles were retained within LE and lysosomes, lacking the necessary elements for viral membrane fusion and core penetration [13].

In this study, we report a novel potential interaction between the ASFV viral protein E199L and the cellular endosomal proteins NPC1, Lamp1 and Lamp2 by immunoprecipitation. In addition, the E248R protein, was able to immunoprecipitated the cellular proteins NPC1 and Lamp2. In contrast, no interaction was found between any of these proteins with NPC2 or PIKfyve. Furthermore, individual interaction studies of the A, C and I domains of NPC1 were included in this analysis. The C domain of NPC1 was found to be the most important domain interacting with both viral proteins. These results suggest that both viral proteins would require the C domain to interact with NPC1.

Importantly, NPC1 has been demonstrated to be an essential and common partner for successful viral infections of several viruses. An important example is EBOV, in which NPC1 is required for viral cytoplasmic penetration. In particular, the protease-primed glycoprotein (GP) of EBOV [57] is able to interact with the C domain of NPC1, triggering membrane fusion [58] and the absence of the C domain triggers resistance to EBOV infection. It has been already demonstrated that EBOV GP translocation of occurs independently of cholesterol transporting function of NPC1 receptor [59–62]. However, recent evidence has highlighted an important role for cholesterol also for EBOV fusion [63]. Furthermore, it has been shown that Lassa virus, an Arenavirus, also binds to Lamp1 for viral fusion at the endosomes [36].

In addition, there are many studies reporting the importance of these cellular proteins in a number of viral infections such as hepatitis C virus (HCV) that requires a paralog of NPC1 protein named Niemann Pick C1 like 1 (NPC1L1) [26]. NCPC1L1 [64,65], responsible for cholesterol homeostasis and absorption, which is crucial for HCV infection.

Likewise, several publications highlighted the relevance of NPC1 in other viral infections such as Human immunodeficiency virus type-1 (HIV). In addition, inhibition of NPC1 function by the chemical compound U18666A, was also shown to inhibit the infection of Chikungunya, an alphavirus and, several flaviviruses such as Zika Virus (ZIKV), West Nile Virus (WNV), Yellow Fever Virus (YFV) and Dengue Virus (DENV) [25,29,31]. In addition, a study showing an interaction between NPC1 and SARS-CoV-2 Nucleoprotein was recently reported [32]. Taken together, all these studies demonstrate the importance of NPC1 and other endosomal proteins for a variety of RNA or DNA viral families and their role in the viral fusion process, which has to be yet fully characterized.

Indeed, compounds specifically designed to interfere with the interaction between the C domain of NPC1 and EBOV GP, severely impacted ASFV infectivity in Vero cells [44] and porcine macrophages (this study), supporting our observed interaction between the C domain of NPC1 and both E248R and E199L.

Importantly, whereas abrogation of one molecule (NPC1/Lamp1) completely inhibits EBOV or Lassa virus infection [33,36], ASFV infection was only partially inhibited and, in view of our results, could involve the interaction of several endosomal proteins. This could indicate that being a large complex virus, ASFV might have developed alternative cellular pathways to achieve viral fusion to escape the endosome. Indeed, we found that E199L was able to interact with NPC1, Lamp1 and Lamp2. From this perspective, it is plausible that due to its genome size, ASFV could have evolved alternative solutions within its more than 150 ORFs, that allow it to overcome this cellular gene abrogation. Therefore, we hypothesized that other LE membrane-localized cholesterol transporters, such as Lamp1 and Lamp2, may also be exploited by ASFV. In fact, these molecules cooperate with NPC proteins in a slower mechanism for endosomal export of cholesterol [19]. However, it is crucial to determine these cellular targets that could allow the design of new intervention strategies targeting the cellular components required for early ASFV infection.

Our results indicated that antiviral drugs targeting NPC1-viral protein interaction are able to decrease infection. In addition to this pharmacological inhibition, we have shown here that the absence or reduction of NPC1 or Lamp2, respectively, impacts ASFV infection. In addition to decreased infectivity, we observed reduced ASFV replication in NPC1 KO cells. The number of cells harbouring viral factories decreased and those remaining, had altered morphology, smaller size, and fewer contacts between the endosomal membranes and other elements of the viral factory. Like human cytomegalovirus (HCMV; a DNA virus), the constitution of the ASFV viral factory [45], replication and assembly of new progeny probably requires lipid-rich cellular membranes. Thus, intact cellular cholesterol efflux may also be required to facilitate ASFV replication after viral entry.

This work shows for first time a potential interaction of ASFV proteins E199L and E248R with NPC1 cellular protein and associated endosomal proteins, as well as their potential relevance in the ASFV virus biology. We have also disclosed novel interactions of ASFV fusion proteins with Lamp1/Lamp2 proteins, while providing controls of other LE membrane-associated proteins that promote endosomal maturation without any interaction with E248R and/or E199L. In fact, other LE membrane-associated proteins to promote endosomal maturation were negative for the interaction such as PIKfyve.

To assess the importance of these interactions on the ASFV life cycle we performed several experiments with chemical compounds, silencing RNAs and CRISPR-KO cells to show the impact of the reduction of these cellular proteins on ASFV infection at early and late stages of infection. We have reported new data on the altered endosomal exit of ASFV in the absence of NPC1, as we were able to identify incoming viral cores could retained inside endosomes, preventing them from continuing infection.

In summary, this study supports a possible novel function of endosomal proteins NPC1, Lamp1 and Lamp2 in facilitating ASFV infection, based on the interaction of ASFV E248R and E199L proteins with this important endosomal protein NPC1. Interestingly, the C domain of NPC1 was shown to be important for this binding in the late endosome, and further protein structural studies would provide detailed characterization of this interaction.

In conclusion, these findings provide further insight into the molecular interactions underlying viral fusion and pave the way for the elucidation of the role of endosomal proteins, NPC1, NPC2 and Lamp 2 in fusion and early formation of viral replication sites for this and other relevant virus models that will be the subject of further studies.

## Material and methods

### Cells

Vero and Human embryonic kidney cells (HEK293T or 293T) were grown at 37°C, 5% CO_2_ atmosphere culture in complete Dulbecco’s Modified Eagle Medium (DMEM) containing 5 or 10% heat-inactivated fetal bovine serum (FBS) respectively, 1% penicillin-streptomycin (P/S) and 1% Glutamax (Gibco, Gaithersburg, MD, USA). Swine alveolar macrophages were collected by alveolar lavage with phosphate-buffered saline (PBS) as previously described [66] and cultured at 37°C in RPMI medium containing inactivated 10% swine serum, 2 mM l-glutamine, 50 μM 2-mercaptoethanol, 20 mM Hepes and 30 μg/ml gentamycin.

### Viruses and infection

We used the cell culture-adapted and non-pathogenic ASFV isolate Ba71V [67] that is adapted to grow in the Vero cell line. We obtained the recombinant virus (ASFV B54GFP) from parental Ba71V, which expresses GFP as a p54 fusion protein [38] and the recombinant Ba71V-30GFP (BPP30GFP) [68]. ASFV viral stocks were propagated and titrated by plaque assay in Vero cells, as previously described [67]. When the recombinant virus B54GFP was used, green fluorescent plaques were observed 4 days after infection under the fluorescence microscope. For immunofluorescence, ASFV stocks were partially purified using a 40% sucrose cushion in PBS at 68,000 × g for 50 min at 4°C and were further used at a MOI of 1 unless otherwise indicated.

### Compounds studied

U18666A (Sigma) is a chemical compound described to target NPC1 function by binding to the NPC1 sterol sensing domain [61]. It has been widely used to interfere with the endosomal cholesterol efflux, mimicking the phenotype observed in Niemann-Pick type C disease [69,70]. Cytotoxicity in Vero cells was previously tested using CellTiter 96 (Promega). Vero cells were pre-treated with DMSO or with increasing concentrations of the drug U18666A ranging from 0.01 to 10 μM, 16h before infection without washing leaving compound during the infection.

All the compounds tested in this work have a purity ≥95% by HPLC and were synthesized at Centro de Investigaciones Biológicas Margaritas Salas (CIB-CSIC) following described procedures. All these molecules belong to the Medicinal and Biological Chemistry (MBC) library and were previously characterized as potential inhibitors of the protein-protein interaction between NPC1 and EBOV glycoprotein (EBOV-GP) [44]. The compounds tested in this study were resuspended at 50 mM in DMSO. Working concentrations of compounds were determined by cytotoxicity assays.

### Cytotoxicity assays

Macrophages were seeded in 96-well plates and incubated with DMEM containing each compound at concentrations ranging from 0 to 100 μM. After 24 hours, cell viability was measured by Cell Titer 96 AQueous Non-Radioactive Cell Proliferation Assay (Promega) following the manufacturer´s instructions. Absorbance was measured at 490 nm using an ELISA plate reader.

Cell viability was reported as the percentage of absorbance in treated cells relative to DMSO- treated cells. The 50% cytotoxic concentration (CC_50_) was calculated and non-toxic working concentrations (over 80% cell viability) were chosen to test the activities of these compounds on porcine macrophages.

### Plasmids and constructs

Part of the methodology used in this section of the study (Fig 1A) was previously described [71]. To generate the ASFV BA71-E248R or E199L with N-terminal EGFP tag (EGFP E248R and EGFP E199L), a codon optimized cDNA sequence for the ORF of the ASFV BA71 (NCBI reference sequence number: NC_001659) was cloned into the pEGFP-C1 (by GeneArt-Thermo Fisher Scientific). Once cloned, the sequence of the plasmids EGFP E248R and EGFP E199L were confirmed by sequencing (Gene Art–Thermo Fisher Scientific).

The Sus scrofa NPC1 WT plasmid was generated by gene synthesis (GeneArt, ThermoFisher) with a gly-gly-gly-ser linker followed by 3x Flags at C terminus end as a tag into pCDNA 3.1+.

To generate single domains plasmids for NPC1, single domain (A, C or I domain) constructs were designed based on the data obtained from Happy Miller et al., 2018, where the domains of NPC1 are described. The A domain from residues 25 to 266; the C domain from residues 373 to 620 and the I domain from residues 854 to 1098. Then, these regions were amplified from the parental plasmid *Sus scrofa* NPC1 WT flanked by HindIII and BamHI restriction sites. The individual domains were then subcloned in a 3.1+ vector with a cassette encoding the following elements: NPC1 signal peptide, the subcloned individual domain, the first transmembrane domain of NPC1, the cytoplasmic C-terminal tail of NPC1 (containing the lysosomal targeting sequence) [72], a gly-gly-gly-ser linker and 3 x Flags as a tag at C-terminal end.

To generate the second set of plasmids used in this study, the pKH3 3xHA plasmid was purchased from Addgene (ref. 12555) and used to clone in frame with the HA tag at the N-terminus the viral proteins E248R, E199L and L1R into the Bam HI and Eco RI restriction sites. In addition, the pHA eIF4E plasmid was purchased from Addgene (ref. 17343) [73].

### Protein expression and transfections in HEK 293T cells

To transfect HEK 293T cells, four 60mm plates were seeded with 3 x10^6^ cells each 24 hours prior to transfection in above described DMEM complete medium. Prior to transfection, the medium was changed to DMEM complete medium at 2% FBS. Transfection of 4 μg of plasmids EGFP E248R or EGFP E199L for each 60 mm dish was then performed using Lipofectamine 2000 (Thermo Fisher Scientific), following the manufacturer instructions. Twenty-four hours post transfection, cells were harvested, lysed and immunoprecipitated using a GFP-Trap kit (Chromotek).

For co-transfections experiments, HEK 293T were seeded under the same conditions described above. Then, 2 μg of the different NPC1 constructs (NPC1, NPC1 A domain, NPC1 C domain or NPC1 I domain) and the viral protein constructs were co-transfected using Lipofectamine 2000 (Invitrogen) following the manufacturer instructions.

### Immunoprecipitations of EGFP-E248R and EGFP-E199L proteins

This part of the methodology was similar to that used in Garcia-Dorival et al., 2021, where EGFP E248R and EGFP E199L immunoprecipitations (IP) were done using a GFP-Trap®_A (Chromotek). To do the IPs, the cell pellet was resuspended in 200 μl of lysis buffer (10 mM Tris HCl pH 7.5; 150 mM NaCl; 0.5 mM EDTA; 0.5% NP40) and then incubated for 30 minutes on ice. The lysate was then clarified by centrifugation at 14000 x *g* and diluted five-fold with dilution buffer (10 mM Tris HCl pH 7.5; 150 mM NaCl; 0.5 mM EDTA). GFP-Trap agarose beads were equilibrated with ice-cold dilution buffer and then incubated with diluted cell lysate overnight at 4°C on a rotator, followed by centrifugation at 2500 x *g* for 2 minutes (min). The bead pellet was washed two times with wash buffer (10 mM Tris HCl pH 7.5; 150 mM NaCl; 0.5 mM EDTA). After removal of the wash buffer, the beads were resuspended in 100 μl of sample buffer Laemmli 2X Concentrate (Sigma Aldrich) and boiled at 95°C for 10 minutes to elute the bound proteins. Buffers used for immunoprecipitations were all supplemented with Halt Protease Inhibitor Cocktail EDTA-Free (Thermo Fisher Scientific).

### Immunoprecipitation of HA viral tagged proteins

Twenty-four hours post-transfection, the cell pellet was resuspended in 200μl of lysis buffer (10mM Tris HCl pH 7.5; 150mM NaCl; 0.5mM EDTA; 0.5%NP40) and incubated for 30 min on ice. The lysate was then clarified by centrifugation at 14000 x g and diluted five-fold with dilution buffer (10mM Tris HCl pH 7.5; 150mM NaCl; 0.5mM EDTA) up to 1ml.

The HA immunoprecipitation was performed using 50μl of the Immobilized Recombinant Protein G Resin (Generon) and specific antibodies against HA (Invitrogen).

In order to perform the pull down, a pre-clearing step was performed by incubating the diluted cell lysate with 50μl of the Immobilized Recombinant Protein G Resin (Generon) on a rotator for 2 hours (h) at 4°C followed by centrifugation at 2500g for 2 min to collect the cell lysate from the beads. Next, 2.5μg of the primary antibody was added into the cell lysate and incubated at 4°C on a rotator for another two h. Then, 50 ul of the protein G resin (Generon) were equilibrated with ice-cold dilution buffer and incubated at 4°C on a rotator with the diluted cell lysate containing the antibody overnight at 4°C on a rotator, followed by centrifugation at 2500g for 2 min to remove non-bounds fractions.

The bead pellet was washed once with wash buffer (10mM Tris HCl pH 7.5; 150mM NaCl; 0.5mM EDTA) and a second time with wash buffer containing a higher salt concentration (10mM Tris HCl pH 7.5; 300mM NaCl; 0.5mM EDTA).

After removal of the wash buffer, beads were resuspended in 100μl of sample buffer, Laemmli 2X Concentrate (Sigma Aldrich) and boiled at 95°C for 10 min to elute bound proteins. The buffers used for the immunoprecipitations were all supplemented with Halt Protease Inhibitor Cocktail EDTA-Free (Thermo Fisher Scientific).

Specifically, for L1R and eIF4E immunoprecipitations, HEK293T cells were seeded in P60 plates the day before transfection and co-transfected with corresponding pCDNA3.1+ 3XFlag and pKH3 3xHA tagged constructions for 24 h. Cells were then lysed in 1ml of ice-chilled lysis buffer consisting of 50 mM Tris, pH 7.4, 150 mM NaCl, 5 mM EDTA, 5% glycerol, 1% Triton X-100 and a protease inhibitor cocktail (complete mini-EDTA free, Sigma), and then, incubated 30 min at 4°C under rotation. The lysates were then centrifuged at 15000 rpm for 30 min at 4°C and incubated with Flag coated beads for 3 hours (h) and 30 min at 4°C under rotation. Finally, the lysates were washed five times with wash buffer (50 mM Tris, pH 7.4, 150 mM NaCl, 5 mM EDTA, 5% glycerol, and 0.1% Triton X-100) and the bound proteins were eluted in 60 μl of Laemmli buffer and boiled at 100°C. Input and pulled down samples were analyzed by western blotting.

### Western blot analysis and antibodies

Pulldowns samples were eluted, boiled in Laemmli buffer and resolved by SDS-PAGE in 7%, 12% or 15% acrylamide-bisacrylamide gels or in Mini-PROTEAN TGX Gels (Bio-Rad) then, the gels were then transferred to a nitrocellulose membrane (Bio-Rad) using the Trans-Blot Turbo Transfect Pack (Bio-Rad) and the Trans-Blot Turbo system (Bio-Rad) and detected with the corresponding antibodies anti-GFP (Santa Cruz Biotechnology), anti-NPC1 (Abcam), anti-NPC2 (Abcam), anti-Lamp1 (BD biosciences), anti-Lamp2 (BD bioscience), anti-PIKfyve (Abnova), anti-Flag (Sigma), anti-HA (Invitrogen), and anti-Tubulin (Sigma), GAPDH (Abcam) or HSP90 (Palex) as loading controls in Western blot (WB) analysis. Anti-mouse IgG (GE Healthcare, Chicago, IL, USA) or anti-rabbit IgG (Bio-Rad) conjugated to horseradish peroxidase was used at a dilution of 1:5000 was used as secondary antibody. Finally, bands obtained after development with ECL reagent were detected on a Molecular Imager Chemidoc XRSplus imaging system. The bands were quantified by densitometry and the data normalized to control values using Image lab software (Bio-Rad).

### Generation of Vero NPC1-knockout by CRISPR/Cas9 technology

We selected the small guide RNA1 (sgRNA): NPC1 KO Fwd: 5´-CACCGCAAACTTGTATCATTCAGAG-3´ and NPC1 KO Rvs: 5´ AAACCTCTGAATGATACAAGTTTGC-3´, among 4 candidate target sequences at the exon 10 genomic region of Vero NPC1 region, designed with the tool Deskgen [74].

The sgRNA was cloned into LentiCRISPRv2 vector according to the manufacturer instructions (GeCKO Lentiviral Crispr toolbox, ZhangLab) and together with the PsPAX2 and VSVg plasmids were transfected in 293T cells (as previously described) grown the previous day in p100 plates to generate the lentiviral particles. Similarly, lentiviral particles were also generated with an empty LentiCRISPRv2 as a control (the selected clone was named as Empty). Lentiviral particles were harvested 72 h after transfection and centrifugated at 4000 rpm for 3 min to remove cellular debris.

The cleared Lentiviral containing medium was transduced in pre-treated Vero cell with Polibrene (8 ug/ml) for 5 min and plated the day before in MW6 plates.

After 72 hours post transduction, the medium was shifted to DMEM 10% FBS with puromycin (20 ug/ml) to select transduced cells. Finally, Vero cells transduced with lentivirus expressing sgRNA1 were selected after checking the absence of NPC1 by WB and indirect immunofluorescence (IFI) with an antibody against NPC1 (Abcam). We were also able to confirm the typical accumulation of cholesterol in late endosomes located in perinuclear position, a common phenotypic change produced under conditions of NPC1 depletion that mimics the Niemann Pick type C disease in CRISPR KO cells. Then, we performed a limited dilution to plate individual clones on 96 MW plate. After clonal expansion for 3 weeks, they were reseeded in MW6 plates, expanded and finally tested and selected by the absence of NPC1 expression by WB compared to controls. Clones were sequenced with the following primers Fwd: 5´-ATATATATGAGCGCTCGCGGCCTGG-3´and Rvs: 5´- GCGCGCCTAGAAATTTAGAAGTCGTT-3´. Of the 31 clones screened, we selected c14, c19, c30 and the c14 was used for the experiments (referred to as NPC1 KO).

### Effect of NPC1 KO on infectivity of pseudotyped VSV

In addition, we confirmed the status NPC1 KO cells by a functional assay in which their resistance to Ebola virus infection using vesicular stomatitis pseudovirions virus with the EBOV glycoprotein. Infection of control Vero parental cells and NPC1 KO cell lines with recombinant VSV (rVSV) pseudotyped with the EBOV glycoprotein (GP) of Mayinga strain or VSV-G was assayed. After 24 h post inoculation, cells were lysed, and luciferase expression was measured in a luminometer as Relative Light Units (RLU). Infectivity percentages were determined by adjusting the number of RLU in Vero cells to 100% for each envelope.

### Flow cytometry analysis

Detection of ASFV infected cells was performed by flow cytometry. Vero cells were infected with recombinant ASFV B54GFP at a MOI of 1 pfu/cell for 16 h. Cells were washed with PBS, harvested by trypsinization, and then washed and collected with flow cytometry buffer (PBS, 0.01% sodium azide, and 0.1% bovine serum albumin). In order to determine the percentage of infected cells per condition, 10,000 cells/time point were scored and analyzed in a FACS Canto II flow cytometer (BDSciences). Infected cell percentages obtained were normalized to values found in control samples.

### Detection and quantitation of the ASFV replication

The quantitation of the number of copies of the ASFV genome was achieved by quantitative real-time PCR (qPCR). DNA from Vero cells and Macrophages infected with ASFV at a MOI of 1 pfu/cell for 16 hpi, was purified using the DNAeasy blood and tissue kit (Qiagen) following the manufacturer’s protocol. DNA concentration was measured using a Nanodrop spectrophotometer. The qPCR assay used fluorescent hybridization probes to amplify a region of the p72 viral gene, as described previously [75]. The amplification mixture was 200 ng of DNA template added to a final reaction mixture of 20 μl containing 50 pmol sense primers, 50 pmol anti-sense primer, 5 pmol of probe and 10 μl of Premix Ex Taq (2×) (Takara). Positive amplification controls were DNA purified from ASFV virions at different concentrations used as standards. Each sample was included in triplicates and values were normalized to standard positive controls. Reactions were performed using the ABI 7500 Fast Real-Time PCR System (Applied Biosystems) with the following parameters: 94°C 10 min and 45 cycles of 94°C for 10 s and 58°C for 60 s.

For quantitation of *Npc1* mRNA levels, RNA was extracted from Vero cells using RNeasy RNA extraction kit (Qiagen) following the manufacturer’s protocol. For retrotranscription QuantiTect Reverse Transcription kit (Qiagen) was used to synthesize cDNA, also following the manufacturer’s protocol. 250 ng of cDNA was used as the template for real- time PCR using the QUANTITECT SYBR GREEN PCR KIT (Qiagen). Reactions were performed using the ABI 7500 Fast Real-Time PCR System (Applied Biosystems). *Npc1* gene expression was normalized to an internal control (18S ribosome subunit), and these values were then normalized against the value of control cells to obtain the fold reduction. The following primers were used: *Npc1*_fwd (5′ GTGTGGTGCTACAGAAAACGG 3′), *Npc1*_rev (5′ AAATGCTGCACTGACAGGGT 3′). The 18S ribosome subunit primers provided in QuantiTect Primer Assay (Qiagen) were used as internal control.

### Silencing shRNA

Lentiviral vectors containing shRNAs to interfere Npc1 (shNPC1) and Npc2 (shNPC2) were purchased from Merk (Darmstadt, Germany). Two different sequences were transduced for shNPC1 and shNPC2:

CCGGGTCCTGGATCGACGATTATTTCTCGAGAAATAATCGTCGATCCAGGACTTTTTTG (TRCN0000418552, sh NPC1 #52)

CCGGAGAGGTACAATTGCGAATATTCTCGAGAATATTCGCAATTGTACCTCTTTTTTTG (TRCN0000421158, sh NPC1 #58)

CCGGCGGTTCTGTGGATGGAGTTATCTCGAGATAACTCCATCCACAGAACCGTTTTTG (TRCN0000293234, shNPC2 #34) CCGGGCTGAGCAAAGGACAGTCTTACTCGAGTAAGACTGTCCTTTGCTCAGCTTTTTG (TRCN0000293236, shNPC2 #36)

CCGGGTACGCTATGAAACTACAAATCTCGAGATTTGTAGTTTCATAGCGTACTTTTTG (TRCN0000298483, shLamp2 #83)

A TRC2 pLKO.5-puro Non-Target shRNA was used as control.

Lentiviral suspensions were prepared in HEK293T. HEK293T were transfected with lentiviral and packaging vectors (psPAX2 and p-CMV-VSV-G) using Lipofectamine 2000 reagent and OPTI-MEM media (ThermoFisher Scientific, Massachusetts, EEUU) for 4h following the manufacturer instructions. The medium was then changed to Iscove’s Modified Dulbecco’s Medium (IMDM) supplemented with 10% FBS, 1% penicillin-streptomycin (P/S) and 1% Glutamax (Gibco, Gaithersburg, MD, USA). The supernatant containing the viral particles was collected after 24 and 48h, centrifugated at 3000 rpm for 5 min to remove cellular debris and filtered with a 0.45-mm filter.

Vero cells were transduced with lentiviral suspensions directly added to the cells.

After 72 hours post transduction, the medium was shifted to DMEM 5% FBS containing puromycin (20 μg/ml) to select transduced cells.

### Indirect immunofluorescence and antibodies, conventional and confocal microscopy

Cells were seeded onto 13 mm glass coverslips in 24 well plates prior to ASFV infection. Cells were then washed with PBS and fixed with 4% paraformaldehyde (PFA) for 15 min. After washing with PBS, the cells were then incubated with 50 mM NH_4_Cl in PBS for 10 min. Then, coverslips were incubated in blocking buffer (0.1% saponin, 0.5% BSA in PBS) for 1 h. Coverslips were then incubated for 1 h in specific primary antibodies diluted in blocking buffer at 37°C. The following rabbit antibodies were used: Rab7 (Cell signaling) and NPC1 (Abcam).

Mouse monoclonal antibodies were: p72 (1BC11, Ingenasa), p150 (17AH2, Ingenasa) and CD63 (Novus biologicals). The appropriate secondary antibody conjugated to Alexa Fluor -488 or -594 (ThermoFisher) was used and cell nuclei was detected with TOPRO3 (ThermoFisher). Coverslips were mounted on glass slides using ProLong Gold (ThermoFisher).

Cells were imaged using a TCS SPE confocal microscope (Leica) with a 63X Oil immersion objective. Image acquisition was performed with a Leica Application Suite Advanced Fluorescence Software (LAS AF). All the images were acquired at a resolution of 1024 X 1024 pixels.

For colocalization analysis, Manders coefficient was used to quantify the degree of overlapping between the staining indicated in the confocal images that were acquired under identical conditions (laser power, pinhole, integration time and gain). The Z-step size between slices was 0.3 microns in each image in all cases and the number of slices or images taken across the structure was equal. The Manders coefficient was quantified with the JACoP (Just Another Colocalization Plugin) plugin for ImageJ software (Wayne Rasband, NIH, Bethesda, USA) as described [76].

To detect free intracellular cholesterol, coverslips were incubated with 100μg/ml filipin (Sigma) for 1h. Filipin signal was recorded using a 390- to 415-nm-wavelength excitation filter and a 450- to 470-nm-wavelength emission filter in a conventional fluorescence microscopy Leica (DM RB). Image analyses were performed with Leica Application Suite advanced fluorescence software (LAS AF) and ImageJ software.

### Viral cores penetration assay

Vero WT, Empty or NPC1 KO cells were plated onto 13 mm glass coverslips in 24 well plates. Cells were infected at MOI of 50 with the Ba71V isolate allowing adsorption at 4° for 90 minutes to allow binding but not endocytosis. Cells were then incubated at 37° with warm media for 3 h to allow virus internalization. Then, cells were washed with cold PBS1X and a brief treatment with trypsin EDTA to remove non-internalized viral particles, washed again and fixed with formaldehyde 4%, permeabilized, blocked and incubated with an anti Rab7 antibody (Cell Signalling) and an anti p150 antibody (17AH2, Ingenasa) to detect LE and viral cores respectively labeled with Alexa secondary antibodies. Finally, the coverslips were mounted on glass slides using ProLong Gold (ThermoFisher). Image acquisition and analysis were performed as explained before. Next, a tailored PlugIn for ImageJ was run to score trapped viral cores within the enlarged Rab7+ vesicles.

The basic workflow consists on reaching both the input directory path where the raw images are located and the path where the outputs will be generated. Once this was done, the tool extracted each series from “.lif” as a multichannel z-stack TIFF format. Next, the “Splitting multi-channel images” plugin splits the RGB stack into its respective red, green and blue components. Each stack was manipulated for extracting each slice as independent. At this point, the processing of viral cores started. First, the segmentation was done to isolate each viral core as a single-particle. To do that, “Auto-Threshold” plugin is called to binarize images through the MaxEntropy method. Then the “Analyze Particles” command was run to count, measure and extract features (morphological, geometrical and descriptive statistics) for each binary viral core. Thereupon, viral cores that meet the filtering conditions were selected for further analysis. Initial filtering isolate masks within an area bigger than four times the pixel size in microns. Next, we only kept masks whose shift on the x-y axis along the slices does not exceed 0.100 microns. From this subset, the mask with the highest mean intensity value was selected. Then, having defined viral cores candidates, we combined them, slice by slice, using the exclusive OR operator to create a composite selection of viral cores.

Next, we started the processing of late endosomes. First, a background correction was applied to correct spatial variations thus obtaining the late endosomes sharpened. Segmentation was performed using the Otsu method. In order to connect separated bright structures, we applied a morphological filtering (closing operator), available under the “MorphoLibJ” library. To get a more accurate analysis, we removed those particles whose area was smaller than 5-fold the pixel size in microns, since this signal biases end-counting results. We then selected areas with a mean intensity value neither 0 nor 255. Selected structures corresponding to late endosomes were combined using the exclusive OR operator. Once selections from both areas of interest (viral cores and late endosomes) were defined, those viral cores which were not intersecting with late endosomes were filtered out using the unary intersection operator “AND” thus selecting those which remained trapped inside these areas.

### Sequence comparison

The sequences of pE248R and pE199L proteins were selected from the NCBI data. NCBI reference sequences for proteins of the different ASFV isolates were: txid10498 for Ba71V, for the Georgia strain txid874269 was used, txid561443 for the South African, txid561444 for Namibia and txid561445 for the Kenyan isolates respectively. For Vaccinia Virus (VACV) Western Reserve (WR) proteins A16L, A26L, G9, and L1R, the NCBI reference: txid10254 was used.

A BLAST was performed against the E199L protein of ASFV Ba71V, finding 25% identity with the A16L fusion protein of Entomopoxvirus Mythimna separata, with NCBI reference: txid1293572. This protein has an identity of 57.3% with VACV WR fusion protein A16L.

Alignments between E199L of ASFV Ba71V and A16L of VACV WR, were performed using the Geneious 6.0.6 program, with a standard BLOSUM62 matrix as these proteins belong to a different viral family. Sequence similarities were found in the alignments, which could originate a structural similarity and possibly a functional homology to be explored. Alignment with the same matrix was performed between the protein L1R of the VACV fusion complex and the ASFV fusion protein E248R.

Also, alignments of the E248R and E199L protein sequences between the different ASFV isolates of were performed using the Geneious 6.0.6 program. The BLOSUM 90 alignment matrix was used, as this is the most accurate comparison matrix between different isolates of the same viral species.

### Statistical analysis

The experimental data were analyzed with Unpaired Student *t*-test by Graph Pad Prism 5 software. Values were expressed in graph bars as mean±sem of at least three independent experiments. Statistical significance was assigned at *p<0.05, **p<0.01, ***p<0.001 and ****p<0.0001; n.s, not significant.

## Supporting information

S1 Fig E248R and E199L structure resemble VACV fusion machinery. (A, B) Identical amino acids are marked green for E248R or purple for E199L. Amino acids conserved 80% are marked in dark grey, those conserved between 60–80% in light grey. Finally, those preserved less than 60% in white. (C-D) Structural comparison with VACV fusion proteins. Myristoylation signal is indicated in red, the three main domains: internal (Int), transmembrane (TM) and external domains (Ext) represented in blocks. Amino acid numbers are indicated. Black lines represent cysteines indicating the positions above and the disulphide bonds in grey. (E) WB of Flag pull-down experiments of 293T cells transiently co-expressing NPC1 Flag together with HA L1R.
(TIF)

S2 Fig E248R and mutants, E199L binding to endogenous NPC1. Membranes used to compose Fig 3B. Dashed boxes were taken to create the western blot composite figure. Membranes were revealed with mouse anti-GFP antibody, rabbit anti-NPC1, rabbit anti-NPC2 and mouse anti-GAPDH antibody.
(TIF)

S3 Fig E248R and mutants, E199L binding to endogenous NPC1. Membranes used to compose Fig 3B. Dashed boxes were taken to create the western blot composition showed in the figure. Membranes were revealed with mouse anti-Lamp1 antibody, mouse anti-Lamp2 antibody and rabbit anti-PIKfyve antibody.
(TIF)

S4 Fig VACV L1R binding to NPC1 and eIF4E as a negative control. (A) Membranes used to compose S1C Fig (B) Membranes used to compose Fig 3C. Dashed boxes were taken to create the western blot composite figure. Membranes were revealed with mouse anti-Flag antibody, mouse anti-HA antibody and mouse anti-tubulin.
(TIF)

S5 Fig Reverse Immunoprecipitation E248R. Membranes used to compose Fig 3C. Dashed boxes were taken to create the western blot composite figure. Membranes were revealed with mouse anti-Flag antibody, mouse anti-HA antibody and mouse anti-tubulin.
(TIF)

S6 Fig Reverse Immunoprecipitation E199L. Membranes used to compose Fig 3D. Dashed boxes were taken to create the western blot composition shown in the figure. Membranes were revealed with mouse anti-Flag antibody, mouse anti-HA antibody and rat anti HSP90.
(TIF)

S7 Fig Chemical inhibitors of NPC1 potently inhibited ASFV infection in primary alveolar macrophages. (A) Cytotoxicity assay at increasing concentrations of the compounds in macrophages. (B) ASFV replication in drug-treated and untreated ASFV infected cells analyzed by real-time PCR. Error bars indicate SD from three independent experiments. Statistically significant differences are indicated by asterisks (*p < 0.05).
(TIF)

S8 Fig NPC1 KO validation in Vero cells. (A) NPC1 detection in Vero or Vero NPC1 KO cell lines (B) Indirect immunofluorescence shows NPC1 in green detected with a specific antibody against NPC1. Scale bar: 25 μm (C-D) NPC1 KO cells depicts dilated endosomes detected in red with the acidic probe Lysotracker. Cholesterol stained with Filipin III (in blue) accumulates in dilated vesicles, similarly as it occurs in cells pre-treated with U18666A drug columns on the right-hand side. Scale bar: 10 μm. (E) Infection of Vero and NPC1-KO-Vero cells with recombinant VSV (rVSV) pseudotyped with Ebolavirus Glycoprotein (EBOV-GP) Mayinga strain or VSV-G 24 h. Cells were lysed 24 h post-infection and assayed for luciferase expression. Percentages of infected cells were determined by setting the number of RLU in Vero cells to 100% for each envelope.
(TIF)

S9 Fig Schematic representation of the workflow of the Image J Plug In to quantify viral cores inside late endosomes. (A) Each series from raw “.lif” files are extracted as single images in TIFF format to be processed. (B) Image pre-processing actions to get discriminate viral cores and late endosome components. (C) Image processing actions to isolate viral cores: segmentation and identification by ID and feature extraction. (D) Selecting viral cores through filtering actions based on a collection of morphological, geometric, statistics and intensity thresholded features. (E) Creating a composite mask combining viral cores through OR operator. (F) Processing of late endosomes applying segmentation, background correction, binary operations as “Fill holes” and morphological filtering using the “closing” operator. (G) Combining late endosomes having mean intensity values neither 0 nor 255 using the exclusive OR operator. (H) Obtaining viral cores which are located inside late endosomes using the conjunction operator AND.
(TIFF)

S10 Fig Viral cores retention within dilated LE in NPC1 KO cells. (A) Representative micrographs from individual slices of ASFV cores detected with an anti-p150 antibody (green) found trapped inside enlarged Rab7+ endosomes labelled with an anti Rab7 antibody (purple) at 3hpi. Scale bar: 10 μm. Scale bar insets: 2 μm.
(TIF)

S1 Data Raw Graphics data. Excel spreadsheet containing, in separate sheets, the underlying numerical data and statistical analysis for Figs 3E, 3F, 4C, 5C, 5D, 6A–6F, 7A–7C, S7 and S8E.
(XLSX)
