# Supplementary figures and images for: New insights into the role of endosomal proteins for African swine fever virus infection
Source: PLoS Pathog. 2022 Jan 26;18(1):e1009784. doi: 10.1371/journal.ppat.1009784 (PMC8820605; doi:10.1371/journal.ppat.1009784)

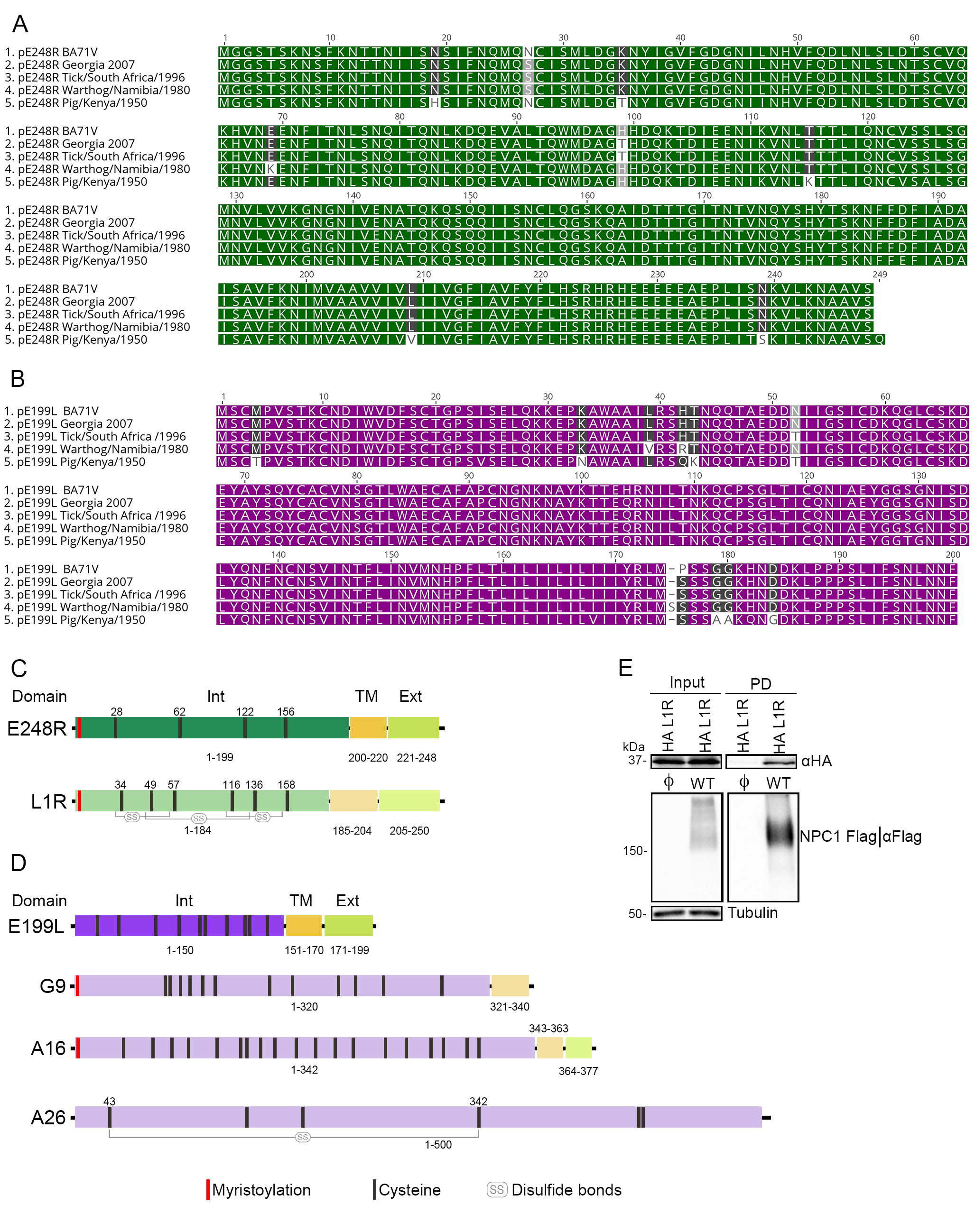

Supplement: S1 Fig — (A, B) Identical amino acids are marked green for E248R or purple for E199L. Amino acids conserved 80% are marked in dark grey, those conserved between 60–80% in light grey. Finally, those preserved less than 60% in white. (C-D) Structural comparison with VACV fusion proteins. Myristoylation signal is indicated in red, the three main domains: internal (Int), transmembrane (TM) and external domains (Ext) represented in blocks. Amino acid numbers are indicated. Black lines represent cysteines indicating the positions above and the disulphide bonds in grey. (E) WB of Flag pull-down experiments of 293T cells transiently co-expressing NPC1 Flag together with HA L1R. (TIF) [file ppat.1009784.s001.tif]

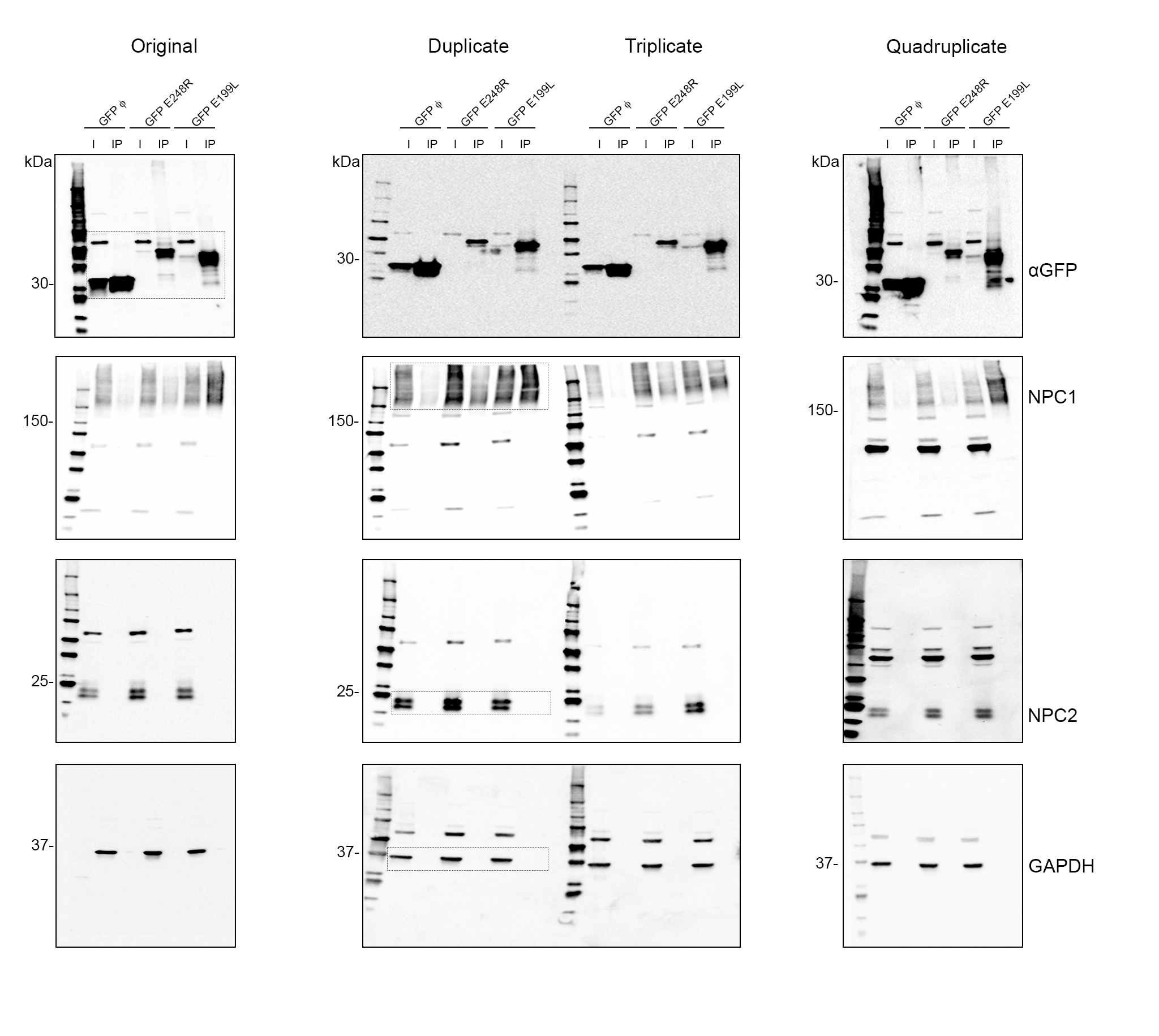

Supplement: S2 Fig — Membranes used to compose Fig 3B. Dashed boxes were taken to create the western blot composite figure. Membranes were revealed with mouse anti-GFP antibody, rabbit anti-NPC1, rabbit anti-NPC2 and mouse anti-GAPDH antibody. (TIF) [file ppat.1009784.s002.tif]

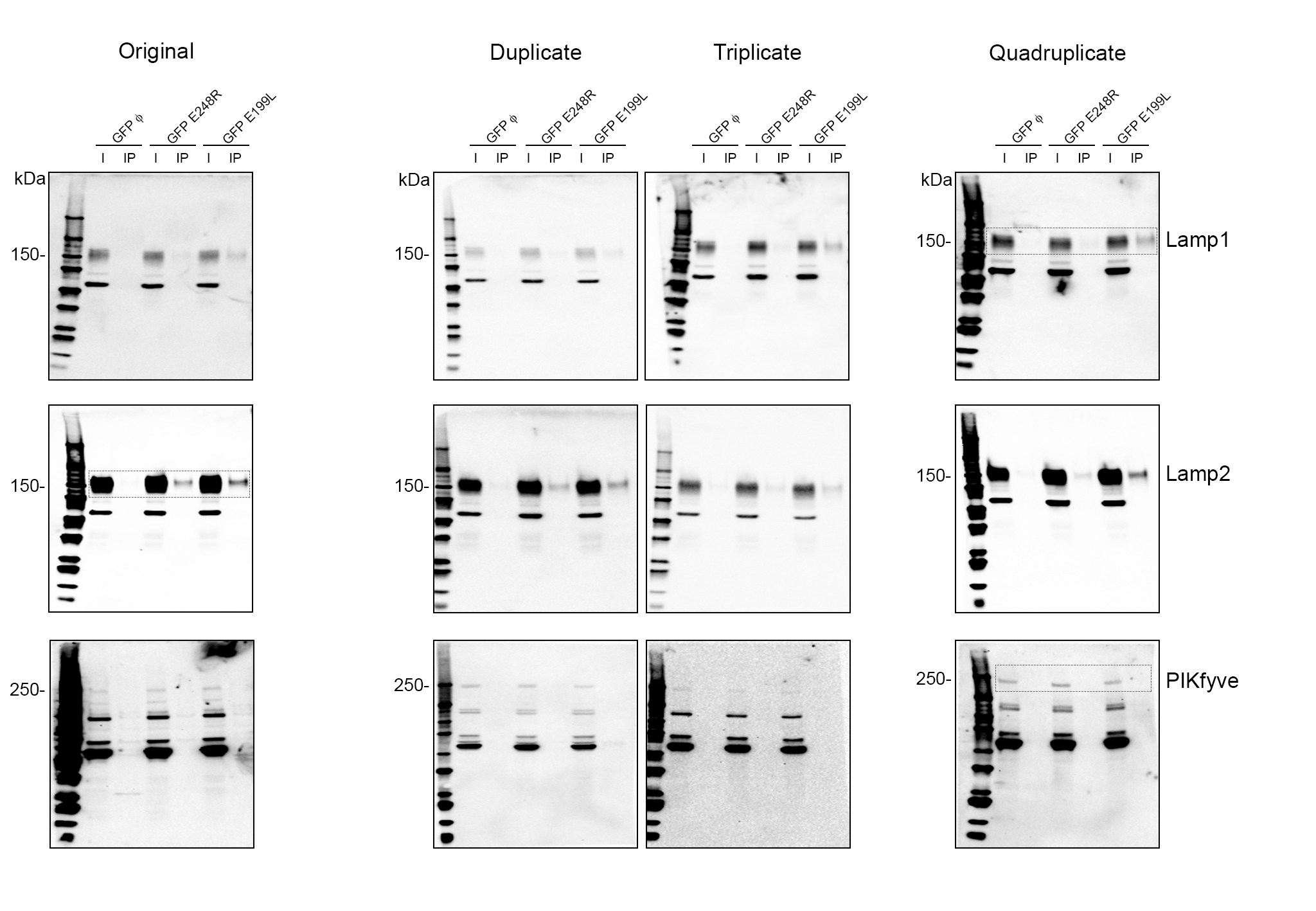

Supplement: S3 Fig — Membranes used to compose Fig 3B. Dashed boxes were taken to create the western blot composition showed in the figure. Membranes were revealed with mouse anti-Lamp1 antibody, mouse anti-Lamp2 antibody and rabbit anti-PIKfyve antibody. (TIF) [file ppat.1009784.s003.tif]

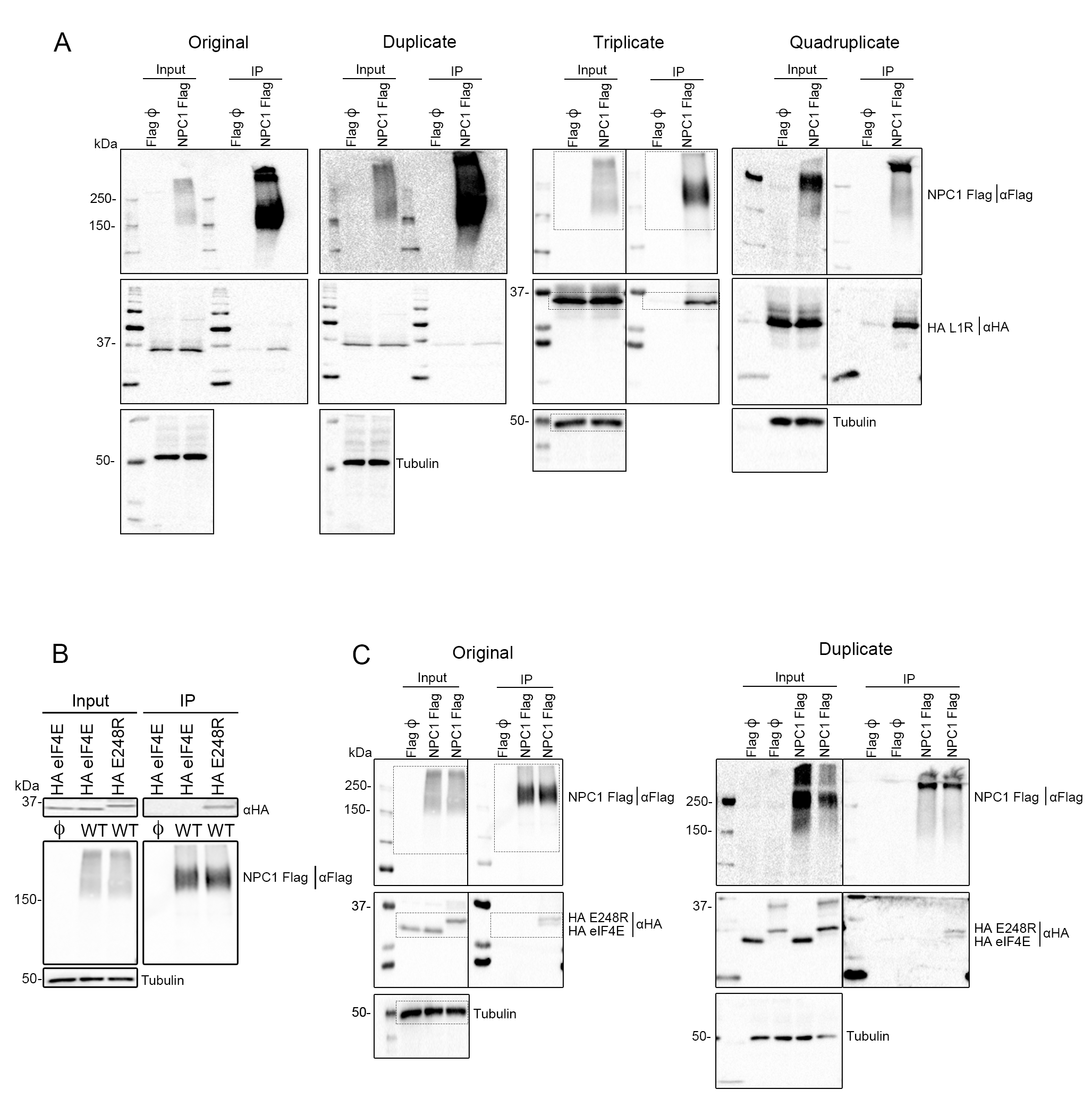

Supplement: S4 Fig — (A) Membranes used to compose S1C Fig (B) Membranes used to compose Fig 3C. Dashed boxes were taken to create the western blot composite figure. Membranes were revealed with mouse anti-Flag antibody, mouse anti-HA antibody and mouse anti-tubulin. (TIF) [file ppat.1009784.s004.tif]

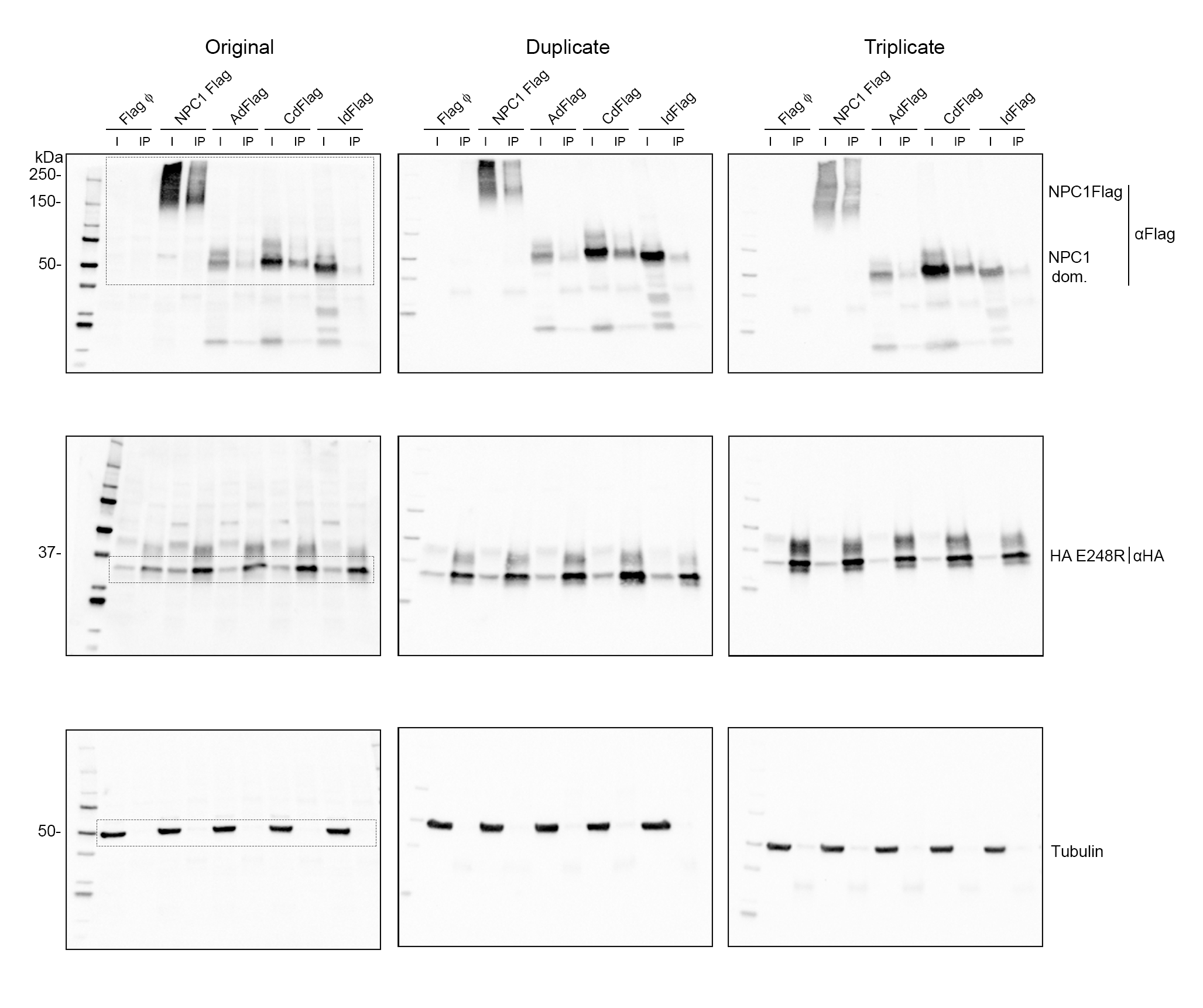

Supplement: S5 Fig — Membranes used to compose Fig 3C. Dashed boxes were taken to create the western blot composite figure. Membranes were revealed with mouse anti-Flag antibody, mouse anti-HA antibody and mouse anti-tubulin. (TIF) [file ppat.1009784.s005.tif]

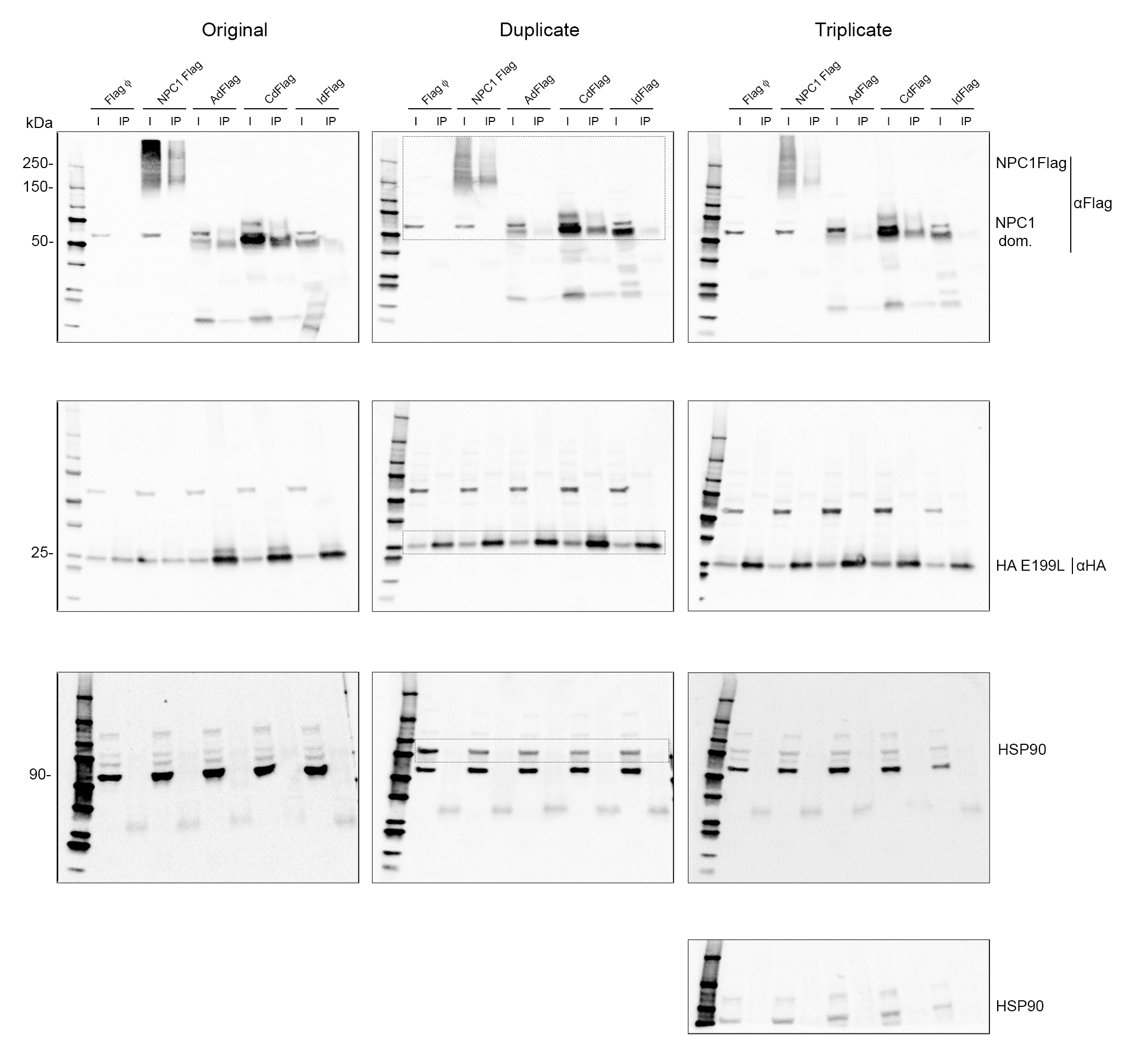

Supplement: S6 Fig — Membranes used to compose Fig 3D. Dashed boxes were taken to create the western blot composition shown in the figure. Membranes were revealed with mouse anti-Flag antibody, mouse anti-HA antibody and rat anti HSP90. (TIF) [file ppat.1009784.s006.tif]

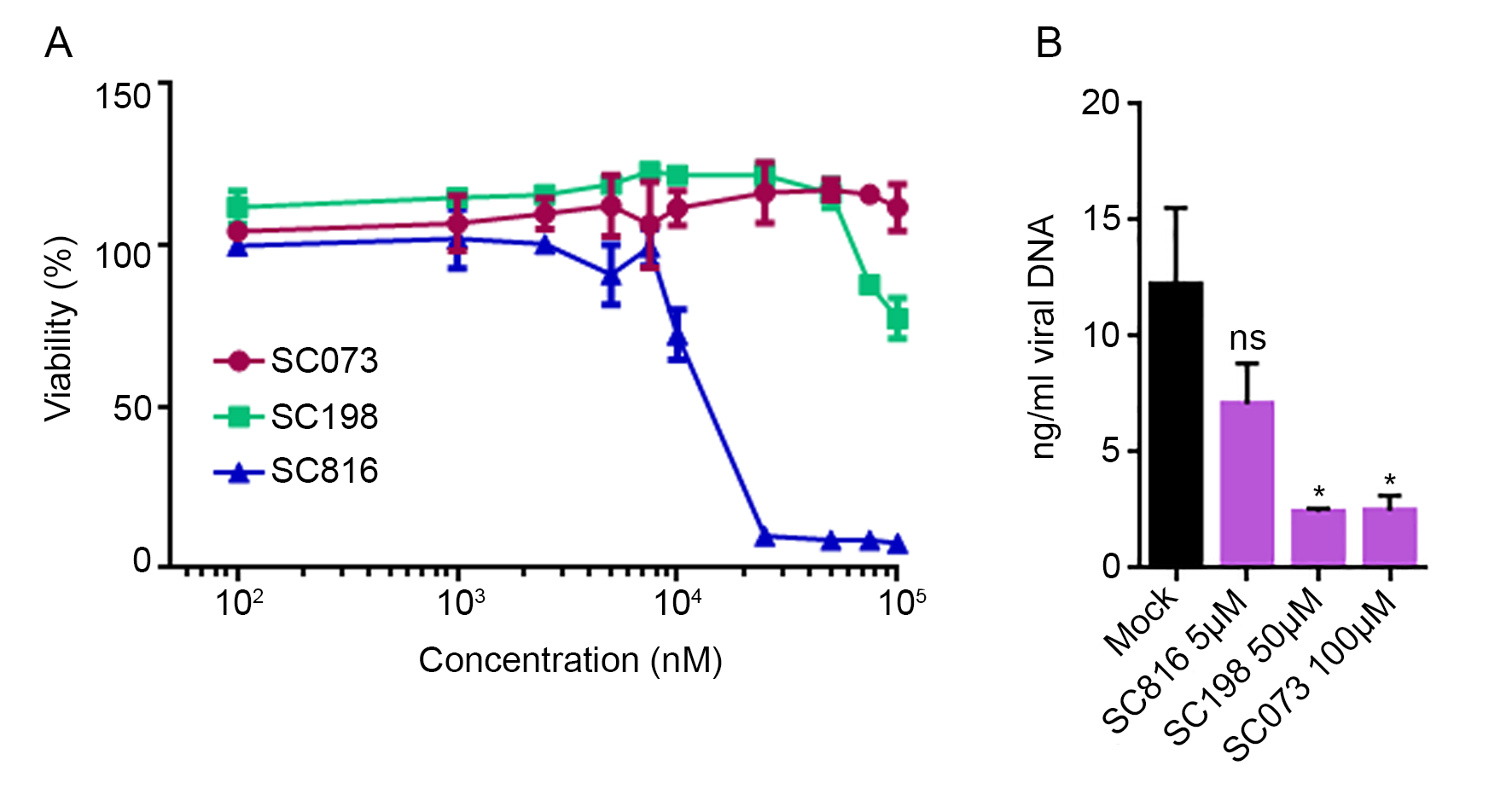

Supplement: S7 Fig — (A) Cytotoxicity assay at increasing concentrations of the compounds in macrophages. (B) ASFV replication in drug-treated and untreated ASFV infected cells analyzed by real-time PCR. Error bars indicate SD from three independent experiments. Statistically significant differences are indicated by asterisks (*p < 0.05). (TIF) [file ppat.1009784.s007.tif]

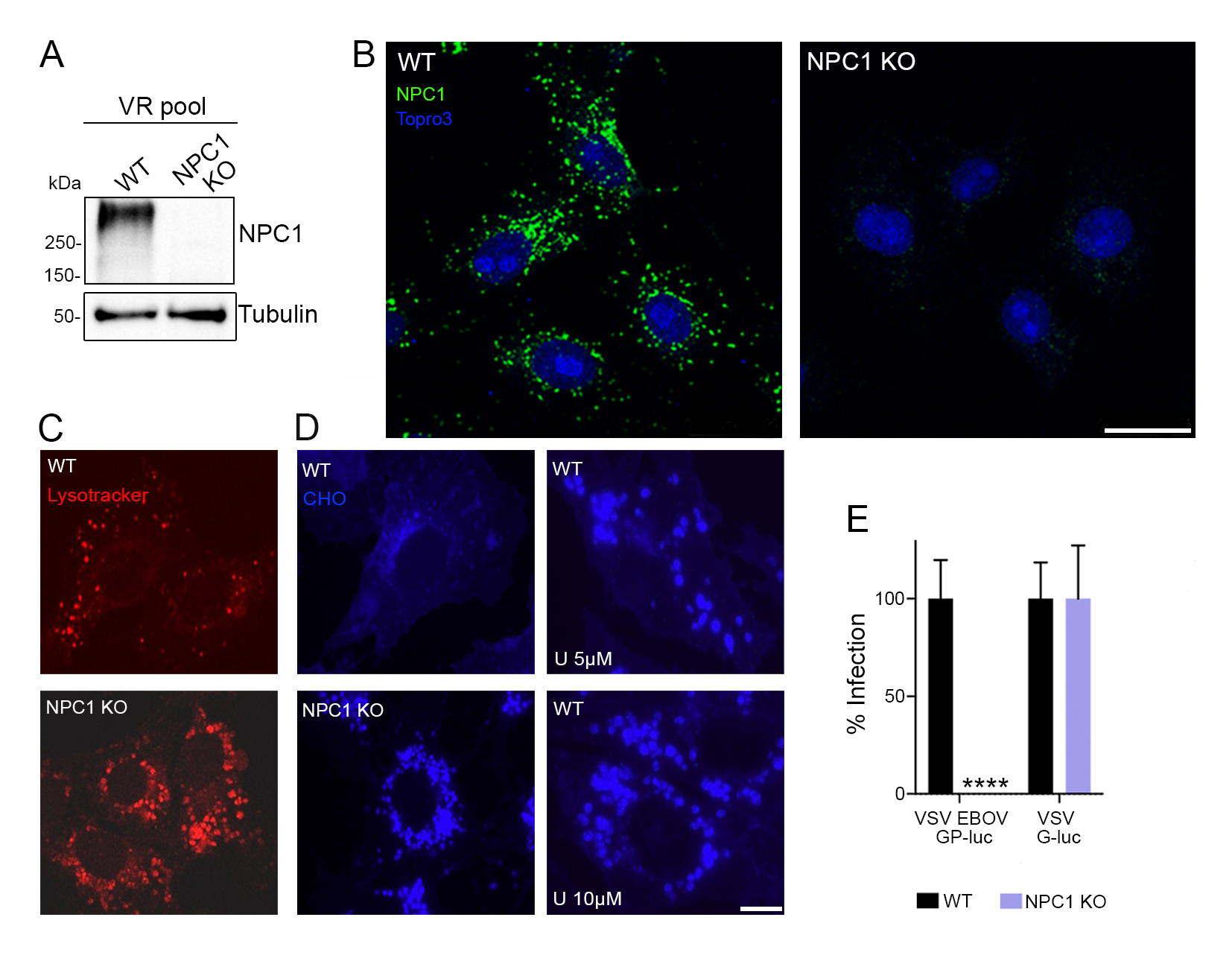

Supplement: S8 Fig — (A) NPC1 detection in Vero or Vero NPC1 KO cell lines (B) Indirect immunofluorescence shows NPC1 in green detected with a specific antibody against NPC1. Scale bar: 25 μm (C-D) NPC1 KO cells depicts dilated endosomes detected in red with the acidic probe Lysotracker. Cholesterol stained with Filipin III (in blue) accumulates in dilated vesicles, similarly as it occurs in cells pre-treated with U18666A drug columns on the right-hand side. Scale bar: 10 μm. (E) Infection of Vero and NPC1-KO-Vero cells with recombinant VSV (rVSV) pseudotyped with Ebolavirus Glycoprotein (EBOV-GP) Mayinga strain or VSV-G 24 h. Cells were lysed 24 h post-infection and assayed for luciferase expression. Percentages of infected cells were determined by setting the number of RLU in Vero cells to 100% for each envelope. (TIF) [file ppat.1009784.s008.tif]

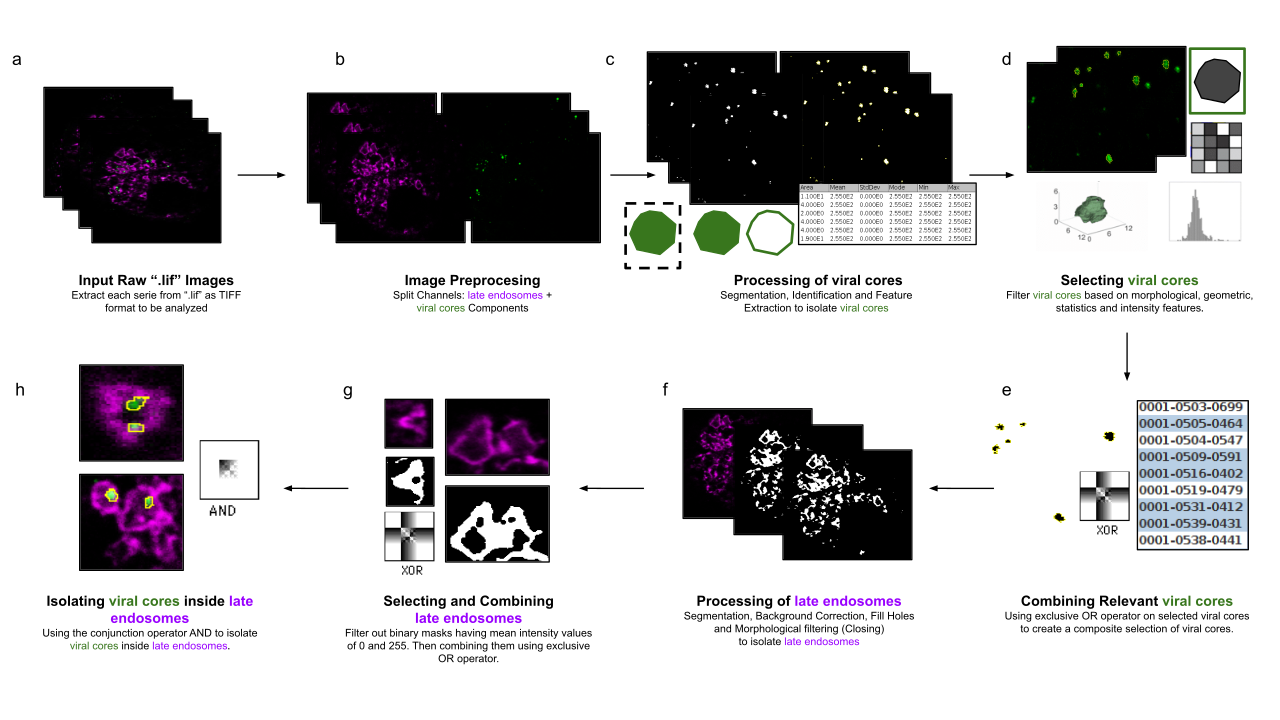

Supplement: S9 Fig — (A) Each series from raw “.lif” files are extracted as single images in TIFF format to be processed. (B) Image pre-processing actions to get discriminate viral cores and late endosome components. (C) Image processing actions to isolate viral cores: segmentation and identification by ID and feature extraction. (D) Selecting viral cores through filtering actions based on a collection of morphological, geometric, statistics and intensity thresholded features. (E) Creating a composite mask combining viral cores through OR operator. (F) Processing of late endosomes applying segmentation, background correction, binary operations as “Fill holes” and morphological filtering using the “closing” operator. (G) Combining late endosomes having mean intensity values neither 0 nor 255 using the exclusive OR operator. (H) Obtaining viral cores which are located inside late endosomes using the conjunction operator AND. (TIFF) [file ppat.1009784.s009.tiff]

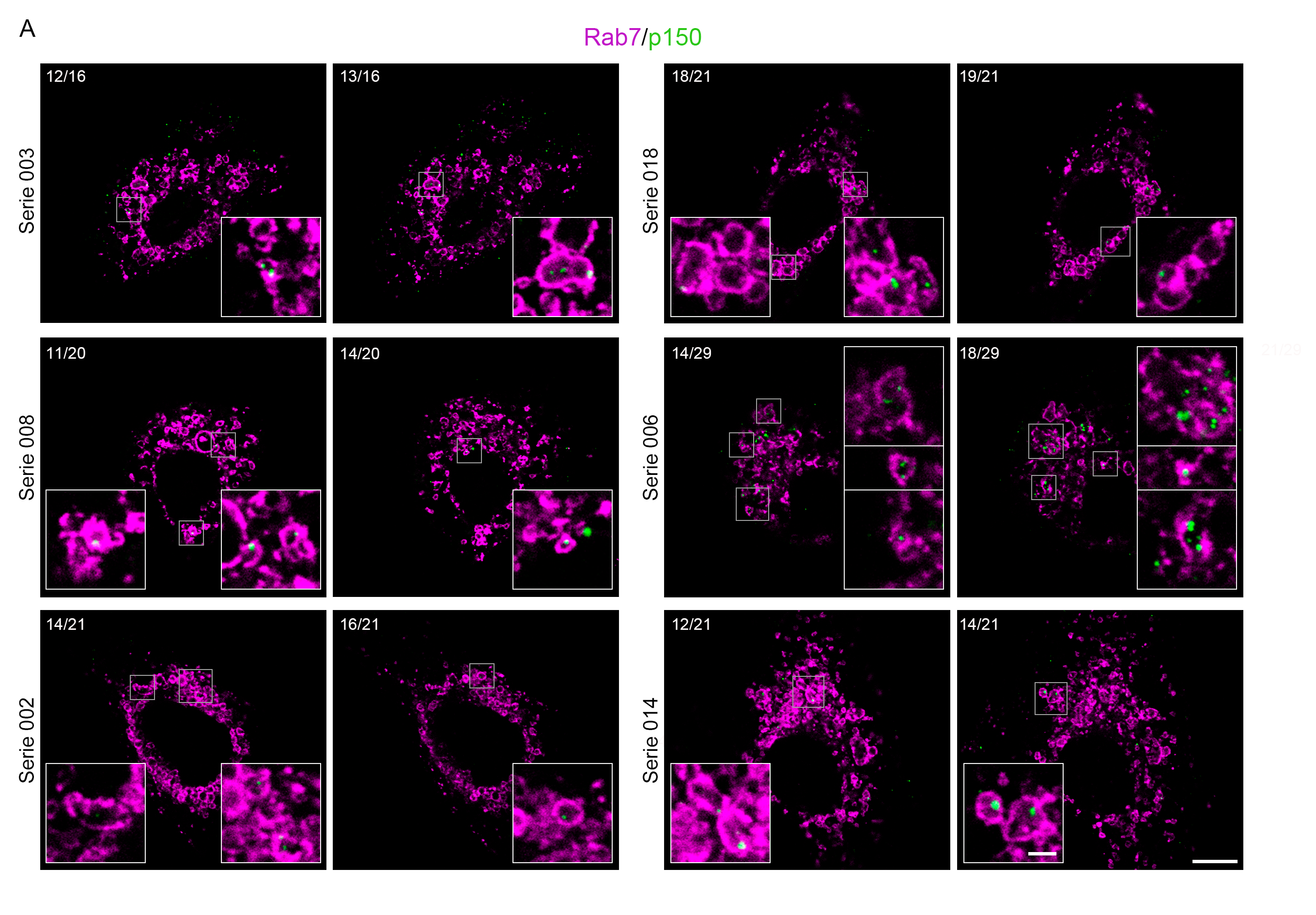

Supplement: S10 Fig — (A) Representative micrographs from individual slices of ASFV cores detected with an anti-p150 antibody (green) found trapped inside enlarged Rab7+ endosomes labelled with an anti Rab7 antibody (purple) at 3hpi. Scale bar: 10 μm. Scale bar insets: 2 μm. (TIF) [file ppat.1009784.s010.tif]
